# The RAS-Effector Interface: Isoform-Specific Differences in the Effector Binding Regions

**DOI:** 10.1371/journal.pone.0167145

**Published:** 2016-12-09

**Authors:** Hossein Nakhaeizadeh, Ehsan Amin, Saeideh Nakhaei-Rad, Radovan Dvorsky, Mohammad Reza Ahmadian

**Affiliations:** Institute of Biochemistry and Molecular Biology II, Medical Faculty of the Heinrich-Heine University, Düsseldorf, Germany; Hungarian Academy of Sciences, HUNGARY

## Abstract

RAS effectors specifically interact with the GTP-bound form of RAS in response to extracellular signals and link them to downstream signaling pathways. The molecular nature of effector interaction by RAS is well-studied but yet still incompletely understood in a comprehensive and systematic way. Here, structure-function relationships in the interaction between different RAS proteins and various effectors were investigated in detail by combining our *in vitro* data with *in silico* data. Equilibrium dissociation constants were determined for the binding of HRAS, KRAS, NRAS, RRAS1 and RRAS2 to both the RAS binding (RB) domain of CRAF and PI3Kα, and the RAS association (RA) domain of RASSF5, RALGDS and PLCε, respectively, using fluorescence polarization. An interaction matrix, constructed on the basis of available crystal structures, allowed identification of hotspots as critical determinants for RAS-effector interaction. New insights provided by this study are the dissection of the identified hotspots in five distinct regions (R1 to R5) in spite of high sequence variability not only between, but also within, RB/RA domain-containing effectors proteins. Finally, we propose that intermolecular β-sheet interaction in R1 is a central recognition region while R3 may determine specific contacts of RAS *versus* RRAS isoforms with effectors.

## Introduction

RAS family proteins, including HRAS, KRAS, NRAS, RRAS1, RRAS2 (or TC21), RRAS3 (or MRAS) and ERAS, act as signaling nodes and regulate the function of various effectors with divergent biochemical functions in all eukaryotes [1,2,3]. Signal transduction implies physical association of these proteins with a spectrum of functionally diverse downstream effectors, *e*.*g*., CRAF, PI3Kα, RALGDS, PLCε and RASSF5, and their activation [1,4,5,6,7,8,9,10]. CRAF, a serine/threonine kinase, activates the MEK-ERK axis and controls gene expression and cell proliferation [11]. PI3Kα generates phosphatidylinositol (3,4,5)-trisphosphate (PIP_3_) and regulates cell growth, cell survival, cytoskeleton reorganization, and metabolism [12]. RALGDS links RAS with RAL, a RAS-related protein, and regulates cellular processes, such as vesicular trafficking and migration [13]. PLCε generates two second messengers of diacylglycerol (DAG) and inositol trisphosphate (IP_3_) leading to an intracellular increase of calcium levels, which controls endocytosis, exocytosis, and cytoskeletal reorganization [14]. RASSF5 forms a complex with MST1/2 kinases, human orthologues of Hippo, and WW45 which promote apoptosis and cell cycle arrest [15].

Gain-of-function RAS mutations are frequently found in human cancers, (*e*.*g*., pancreatic cancer [16]) and developmental disorders, including Noonan syndrome [17,18,19]. Whereas the latter is thought to be commonly caused by dysregulation of mainly one pathway, the RAS-MAPK pathway [19], RAS-mediated cancer progression involves activation of several pathways, *e*.*g*., PI3K-AKT [3,20], RALGDS-RAL [9,13], PLCε-second messengers [14] or Hippo-YAP [21] as well as RAS-MAPK [22]. Understanding how effectors selectively recognize RAS-GTP is an attractive approach to functionalize peptides and peptidomimetics capable of inhibiting RAS interactions and signaling.

**Fig 1:**
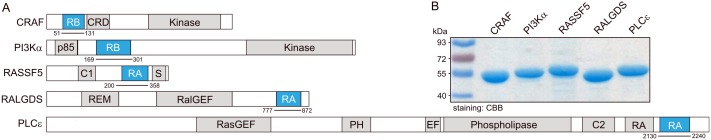
Domain organization of RAS effectors and different proteins used in this study. (A) Various domains are highlighted, including RAS association domain (RA) and RAS-binding (RB) domain in blue. The numbers indicate the N- and C-terminal amino acids of the respective effector domain used in this study. Other domains are: C1, cysteine-rich lipid binding; C2, calcium-dependent lipid binding; CRD, cysteine rich domains; DEP, Dishevelled/Egl-10/Pleckstrin; EF, EF-hand; kinase, serine/threonine or phosphoinositide kinase; PH, pleckstrin homology; PI3K, Phosphoinositide 3-kinase family, accessory *domain;* PP, proline-rich region; RA, RAS association; RALGEF, RAL specific guanine nucleotide exchange factor; RASGEF, RAS specific guanine nucleotide exchange factor; RB, RAS binding; REM, RAS exchanger motif; SARAH, Salvador/RASSF/Hippo. (B) Coomassie brilliant blue (CBB) stained SDS-PAGE of purified MBP fusion proteins used in this study.

RAS effectors contain either a RAS binding (RB) or a RAS association (RA) domain (among other domains; Fig 1) [7,23,24]. RAS-effector interaction essentially requires RAS association with membranes [25] and its activation by specific regulatory proteins (*e*.*g*., guanine nucleotide exchange factors or GEFs), leading to the formation of GTP-bound, active RAS [26,27,28]. Notably, RAS proteins change their conformation mainly at two highly mobile regions, designated as switch I (residues 30–40) and switch II (residues 60–68) [29,30]. Only in GTP-bound form, the switch regions of the RAS proteins provide a platform for the association with effector proteins, especially through their RB or RA domains, respectively. This interaction appears to be a prerequisite for effector activation [24,31,32,33]. RB/RA associations with RAS proteins do not exhibit the same mode of interaction among different RAS effectors [24,34,35,36]. However, CRAF-RB and RALGDS-RA domains share a similar ubiquitin-like fold and contact the switch I region *via* a similar binding mode. In contrast, PI3Kα-RB, RASSF5-RA and PLCε-RA domains do not share sequence and structural similarity but commonly associate with the switch regions, especially switch I [34,35,36,37,38]. Early cell-based studies have shown that distinct amino acids in switch I, *e*.*g*., Thr-35, Glu-37, Asp-38 or Tyr-40) dictate effector specificity [39,40,41,42]. However, there is no clear explanation for such a differential selection of the switch I region by various effectors.

To date, various methods and different conditions for measuring the binding affinity between different effectors and RAS proteins, especially HRAS, have been used in many laboratories (reviewed in [4,24,43]), as summarized in Table 1. In this study, the interactions of five different RAS proteins with both the RB domains of CRAF and PI3Kα, and the RA domains of RALGDS, PLCε and RASSF5 were reinvestigated under comparable conditions using fluorescence polarization. In addition, available complex structures and sequence alignments were utilized to construct an interaction matrix and systematically assess the association of investigated effector domains with various RAS proteins. The dissociation constants (K_d_ values) obtained were combined with the interaction matrix enabling us to determine common hotspots as critical specificity-determining residues and to predict selectivity of five RB- and RA-containing proteins.

**Table 1:** Register of dissociation constants (K_d_) determined for the RAS-effector interactions.

RAS	Nnucleotide^a^	Effectors^b^	K_d_ (μM)	Method^c^	T (°C)	Reference
HRAS	mGTPγS	CRAF-RB	0.005	GDI	37	[102]
	mGDP	CRAF-RB	24.0	GDI	37	[102]
	[^3^H]GTP	CRAF-RB	0.065	SPA	37	[103]
	[γ^32^P]GTP	CRAF-N275	0.029	CPA	4	[104]
	[γ^32^P]GTP	RALSGDS-C127	0.028	CPA	4	[104]
	mGppNHp	AF6-RA1	2.4	GDI	37	[105]
		AF6-RA1	2.4	FK	10	[106]
		AF6-RA1	2.8	FK	25	[107]
		CRAF-RB	0.16	FK	25	[107]
		CRAF-RB	0.14	FP	25	[108]
		CRAF-RB	0.22	FP	25	[18]
		CRAF-RB	0.018	GDI	37	[102]
		CRAF-RB	0.16	GDI	25	[109]
		CRAF-RB	0.33	GDI	25	[110]
		RALGDS-RA	2.70	FP	25	[108]
		RALGDS-RA	1.30	FK	25	[107]
		RALGDS-RA	3.50	GDI	37	[111]
		RASSF5-RA	5.20	FP	25	[108]
		RASSF5-RA	0.8	GDI	37	[35]
		RASSF5-RA	0.08	FK	37	[35]
		PLCε-RA2	5.20	FP	25	[108]
	GppNHp	CRAF-RB	0.08	ITC	25	[112]
		AF6-RA1	3.00	ITC	25	[112]
		AF6-RA1	2.20	ITC	25	[24]
		RALGDS-RA	1.0	ITC	25	[112]
		RALGDS-RA	1.0	ITC	25	[24]
		RASSF1-C1-RA	39.0	ITC	25	[24]
		RASSF5-C1-RA	0.40	ITC	25	[113]
		RASSF5-RA	0.21	ITC	25	[113]
		PLCε-RA2	0.82	ITC	25	[24]
		PLCε-RA1/2	0.98	ITC	25	[24]
		AF6-RA1(Y32W)	0.58	WF	10	[106]
KRAS	mGppNHp	CRAF-RB	0.04	GDI	37	[102]
		CRAF-RB	0.102	ITC	25	[17]
	GppNHp	CRAF-RB	0.056	BBA	25	[114]
NRAS	mGppNHp	RAF-RB	0.04	GDI	37	[102]
		PI3Kγ-RB	2.90	FP	20	[36]
RRAS1	mGppNHp	CRAF-RB	252.9	FP	25	[115]
		RALGDS-RA	376.7	FP	25	[115]
		RASSF5-RA	54.6	FP	25	[115]
		PLCε-RA1	306.6	FP	25	[115]
		PI3Kα-RB	330.5	FP	25	[115]
		CRAF-RB	1.10	GDI	37	[116]
RRAS3	GppNHp	AF6-RA1	2.80	ITC	25	[24]
		RALGDS-RA	3.70	ITC	25	[24]
		PLCε-RA1/2	7.50	ITC	25	[24]

^a^ Different GTP or GDP analogs bound to HRAS have been used: GppNHp, Guanosine-5'- [(β,γ) -imido]triphosphate; mGDP, N-methylanthraniloyl-guanosine-5'-diphosphate; mGppNHp, N-methylanthraniloyl-GppNHp; mGTPγS, N-methylanthraniloyl-guanosine 5'-[gamma-thio-]triphosphate; [^3^H]GTP, tritium-labeled GTP; [γ^32^P]GTP, gamma 32-phosphate-labeled GTP.

^b^ RAS binding (RB) and RAS association (RA) of various effectors were used; CRFA-N275 contains the N-terminal 275 aa encompassing RB domain; RALGDS-C127 contains the C-terminal 127 aa encompassing RA domain. PI3Kγ-RB consists of aa 144–1102.

^c^ BBA, bead–based assay; CPA, co-precipitation assay; FK, fluorescence kinetics; FP, fluorescence polarization; GDI, guanine nucleotide dissociation inhibition; ITC, isothermal titration calorimetry; SPA, scintillation proximity assay; SPR, surface plasmon resonance.

## Materials and Methods

### Constructs

Fragments of human genes encoding both RBs of CRAF (accession number P04049; amino acids or aa 51–131), PI3Kα (P42336; aa 169–301), and RAs of RALGDS (Q12967; aa 777–872), PLCε (Q9P212; aa 2130–2240), RASSF5 (Q8WWW0; aa 200–358) were cloned into pMal-c5X-His vector. Constructs for the expression of human HRAS, KRAS, NRAS, RRAS1 and RRAS2 isoforms were described previously [5].

### Proteins

All RAS and the effector proteins were expressed in *Escherichia coli* using the pGEX and pMAL-His expression systems and prepared using glutathione and Ni-NTA based affinity chromatography as described previously [18]. RAS-mGppNHp was prepared as described [18].

### Fluorescence polarization

RAS-effector interaction was performed in 50 mM Tris/HCl pH 7.5, 100 mM NaCl, 5 mM MgCl_2_ and 3 mM dithiothreitol at 25°C using a Fluoromax 4 fluorimeter in polarization mode as described [18]. Increasing amounts of MBP-tagged effector proteins (0.05–100 μM) titrated to 1 μM RAS-mGppNHp resulted in an increase of polarization. Equilibrium dissociation constants (K_d_) were calculated by fitting the concentration dependent binding curve using a quadratic ligand binding equation.

### Sequence and structural analysis

Sequence alignments were performed with Bioedit program using the ClustalW algorithm [44]. Chimera was used to adjust sequence alignments with superimposed structures [45]. A python code was written to match sequence alignments with complex structures (S1 Table) and calculate intermolecular contacts in the form of an interaction matrix. The intermolecular contacts were defined as pairs residues with a distance ≤4.0 Å between effectors and RAS proteins in available complex structures in the protein data bank (http://www.pdb.org). Biopython modules [46] were also used to elucidate corresponding residues in all available complex structures. All structural representations were generated using PyMol viewer [47].

## Results

### A general approach for quantitative study of RAS-effector interaction

As previous studies focused mainly on HRAS interaction with effectors, there is a lack of information for other RAS proteins (Table 1). Dissociation constants (K_d_ values) have been invaluable in providing insights into particular RAS-effector interactions. However, they have been obtained under various conditions using diverse experimental techniques (see Table 1) and cannot be used as such for a comparative evaluation of the interaction of different RAS proteins with various effectors. For this reason, we set out to analyze the interaction of HRAS, KRAS, NRAS, RRAS1 and RRAS2, with five distinct RB- and RA-containing effectors (Fig 1) under the same conditions. Since the kinetic analysis using stopped-flow spectrofluorometric method was not applicable to all isolated effector proteins, we utilized the fluorescence polarization approach [48].

**Fig 2:**
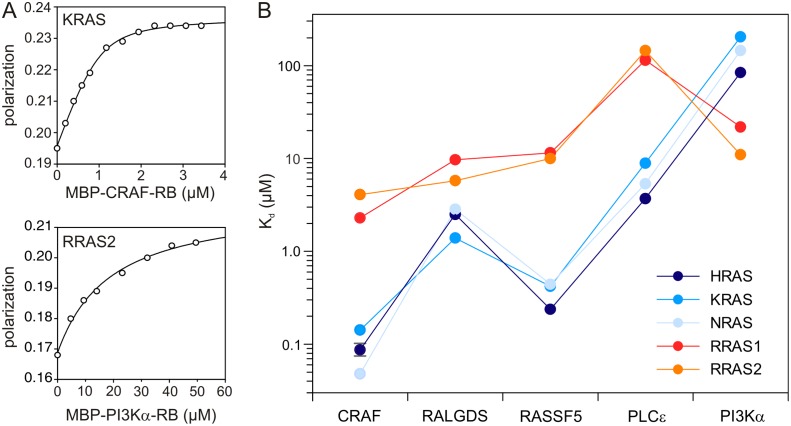
Equilibrium dissociation constants for RAS-effector interaction determined Fluorescence polarization. (A) Fluorescence polarization experiments were conducted by titrating mGppNHp-bound, active forms of RAS proteins (1 μM, respectively) with increasing concentrations of the respective effector domains as MBP fusion proteins. Data of two representative experiments for the interaction of KRAS (upper panel) and RRAS2 (lower panel) with CRAF-RB and PI3Kα-RB, respectively, are shown. All other data are illustrated in S1 Fig (B) Evaluated equilibrium dissociation constants (K_d_) in μM shown as data points illustrate a significant difference in the binding properties of the effector proteins with both RAS and RRAS isoforms, respectively. A mean value of 0.94 ± 0.014 μM has been determined for the interaction between HRAS and CRAF to exemplify the reproducibility of this approach.

Therefore, we prepared both, the RAS proteins in complex with mant (m) GppNHp, a non-hydrolysable fluorescent GTP analog, and the effector proteins fused to maltose-binding protein (MBP, 42 kDa). We chose the MBP because it increases the molecular mass of small-sized RB or RA domains, leads to an amplified fluorescence signal (Fig 2A) and ensures a homogeneous monomeric form of the fusion proteins. GST-fusion protein in contrast yielded a mixture of dimeric and monomeric species (data not shown). Equilibrium titration experiments revealed sufficient signal changes upon binding and guaranteed comparable experimental conditions for all measurements. By taking advantages of this method, complexes formed between these two types of proteins provided distinct polarized signals (Fig 2A and S1 Fig) that enabled us to determine K_d_ values for RAS-effector interactions (Table 2).

**Table 2:** Dissociation constants (K_d_) in μM for the interaction between RAS proteins and effectors.

Effector domains^a^	HRAS	KRAS	NRAS	RRAS1	RRAS2
CRAF-RB	0.094	0.142	0.048	2.29	4.09
RASSF5-RA	0.238	0.421	0.442	11.5	10.00
RALGDS-RA	2.50	1.39	2.84	9.71	5.78
PLCε-RA2	3.70	8.90	5.36	114.4	145.4
PI3Kα-RB	84.3	204.7	145.0	11.00	18.10

^a^ The effector domain were used in these fluorescence polarization measurement as MBP fusion.

The affinities determined for the interaction between RAS proteins and individual effector domains vary between 48 nM for the NRAS–CRAF interaction and 205 μM for the interaction between KRAS and PI3Kα (Fig 2B; Table 2). In general, the tested RAS proteins can be nicely divided according to their affinities into two distinctive groups, the first comprising HRAS, KRAS, NRAS and the second the RRAS proteins. Highest affinities were obtained for CRAF, which were roughly 3–8 fold higher when compared to those for RASSF5, followed by RALGDS and PLCε with K_d_ values in the lower micromolar ranges (Fig 2B; Table 2). In contrast, RRAS1 and RRAS2 have similar micromolar affinities for the effectors and, interestingly, also for PI3Kα but not for PLCε. Our data clearly support previous findings (see Table 1) that isolated effector domains, such as RB or RA, represent functional units, capable of recognizing and tightly binding to RAS proteins. Exceptions are the low affinity of PLCε RA domain for the RRAS proteins and PI3Kα RB domain for HRAS, KRAS and NRAS.

### Identification of hotspots within protein interfaces

**Fig 3:**
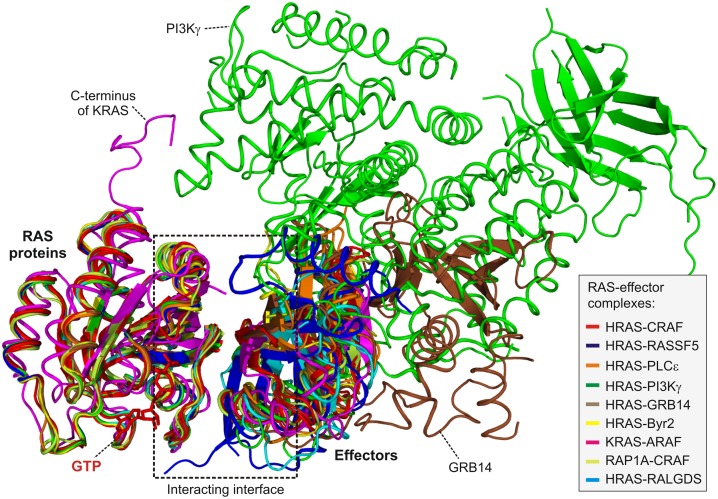
Superposition of all available RAS–effector complex structures. Nine structures of RAS-effector domain complexes, found in a PDB search, including HRAS-CRAF (PDB code: 4g0n, 4g3x, 3kud; red), HRAS-BYR2 (PDB code: 1k8r; yellow), RAP1A-CRAF (PDB code: 1gua; lime), KRAS-ARAF (PDB code: 2mse; magenta), HRAS-RALGDS (PDB code: 1lfd; cyan), HRAS-PI3K (PDB code: 1he8; green), HRAS-PLCε (PDB code: 2c5l; orange), HRAS-RASSF (PDB code: 3ddc; blue), HRAS-GRAB14 (PDB code: 4k81; brown), were overlaid in ribbon presentation. Additional properties outside the interaction interface (box) are indicated.

**Fig 4:**
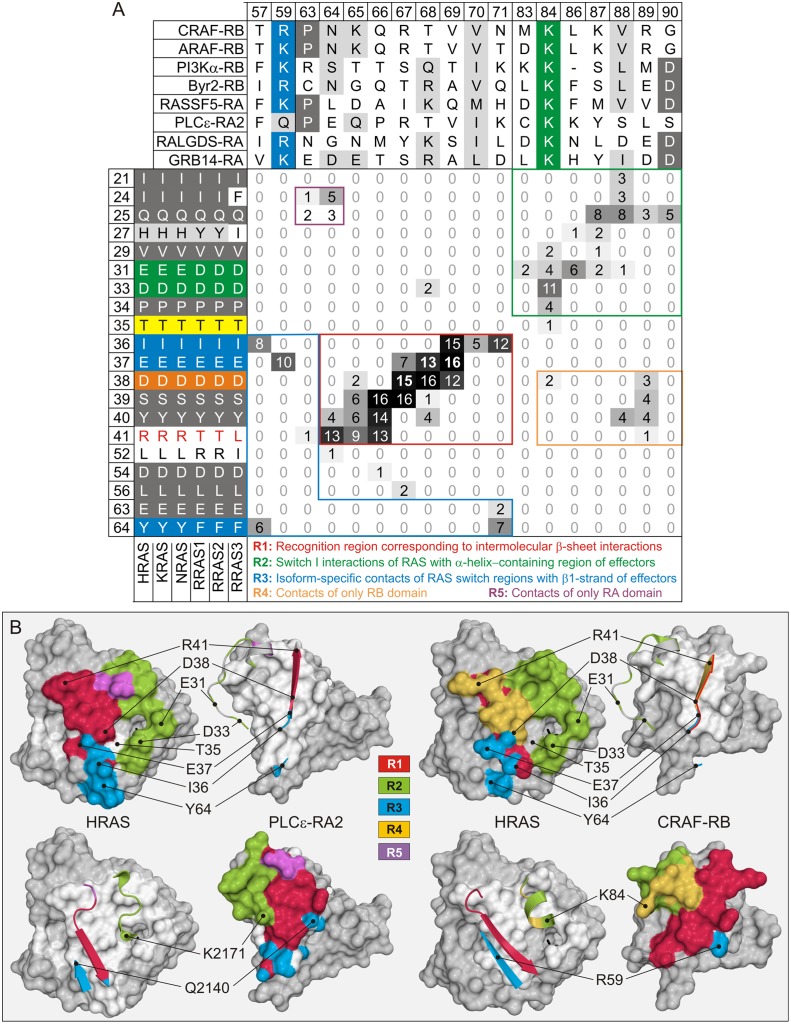
RAS-effector interaction hotspots. (A) Interaction matrix of RAS isoforms and effector proteins. Interaction matrix is launched to demonstrate interaction residues in all available structures (see Fig 3 and S4 Fig). Left and upper parts comprise the amino acid sequence alignments of the RAS proteins and the effector domains, respectively. Each element corresponds to a possible interaction of RAS (row; HRAS numbering) and effector (column; CRAF numbering) residues. As indicated, interaction matrix represents five main regions, which cover the main interacting interfaces. (B) The five main regions, comprising the main hotspot for the RAS-effector interaction, are highlighted as ribbon and surface representations in the corresponding colors for the structures of HRAS-PLCε (PDB code: 2C5L) and HRAS-CRAF (PDB code: 4G0N). Key amino acids which are highlighted by colored background in A are indicated on the structures as well.

To date eleven complex structures of RAS proteins and their effectors has been determined (S1 Table). Since some of them contain more than one complex in the unit cell, there were altogether sixteen complex structures available for the analysis. In order to map atomic interactions responsible for observed variable affinities, we have extracted information about interacting interface from all these complex structures and combined them with their sequence alignments (S2 and S3 Figs). Interestingly, effectors show low sequence similarity (S2A Fig), but their mode of interaction appears to be well conserved as can be seen after a superposition of the complex structures on the RAS structure (Fig 3 and S4 Fig). However, some amino acids aligned according to the sequence were quite distant in the space. Therefore, we edited the sequence alignment to synchronize it with structural alignment (S2A Fig). Our python code finally took sequence alignments with PDB files of complex structures as inputs and calculated all interaction pairs in analyzed complex structures in the form of a matrix (Fig 4A).

### Interaction matrix and binding regions

An interaction matrix relates, in a comprehensive manner, the interacting residues on both sides of complexes, with RAS isoforms as rows and effector proteins as columns (Fig 4A). All numbering in this study is based on HRAS and CRAF proteins. Each element of the matrix accounts for the number of contacts between corresponding residues in all analyzed structures. Residues, involved in at least one interaction, were considered to represent a general interaction interface between RAS proteins and their effectors. Interacting amino acids form continuous patches on both sides of the complexes. Particular modes of interactions between parts of these two patches correspond to regions in the interacting matrix. We identified five such distinct regions (denoted from R1 to R5) in the matrix which had the highest number of interactions. These are separately highlighted in Fig 4.

Most pronounced is R1, located in the middle of matrix. Inspection of the particular interactions corresponding to this region clearly shows an arrangement of intermolecular β-sheet interactions in an anti-parallel fashion (Fig 4B). As many of these contacts in R1 are mediated by main-chain/main-chain interactions, we divided each element of R1 in the matrix into four categories of interactions (main-chain–main-chain, main-chain–side-chain, side-chain–main-chain and side-chain–side-chain; S5 Fig). Main-chain–main-chain interactions typically involve hydrogen bonds between the N-H group and the carbonyl oxygen. We found three interaction hotspots in all RAS-effector complexes, which represent a central recognition site in R1. These amino acids are Glu-37, Asp-38 and Ser-39 from the RAS side and positions 66 to 69 from the effector side (Fig 4A, red box). However, side-chain interactions are also highly populated in these spots indicating that the nature of amino acids in R1 region also influences the RAS-effector association (S5 Fig).

Another distinct region is R2, which corresponds to the interactions between the residues 21 to 34 of RAS, including the N-terminal half of switch I, and an elongated loop containing an α helix (in the case of PLCε and PI3Kα) and two α helices covering positions 83 to 90 (Fig 4). However, the overall shape of corresponding amino acids as well as the spatial orientation of α-helical structures is very diverse (Fig 4B). These structural diversities not only cause widely dispersed interactions in R2, but are also responsible for the interactions in the frames of region R4. The capability of RB domains in R2 to interact also with the β-strand in switch I of RAS simultaneously involves the recognition region R1 and gives rise to the region R4 (Fig 4B; upper panel). On the other hand, the spatial position of the N-terminal residues of RA domains in R1 is similar to the position of the C-terminal residues RB domains in R2 resulting in the interactions established in the region R5. Remarkably, the interaction matrix gives the hints for a region R3 (Fig 4) that could not be defined as a general interaction patch from a direct pair-wise comparison of individual complex structures. This region comprises critical residues, including Ile-36, Glu-37 and Tyr-64 on the RAS side, and positions 57, 59 and 71 on effector side. R3 very likely determines the selectivity of RAS-effector interaction, especially because of sequence deviations at this region (Arg-41 and Tyr-64) when comparing HRAS, KRAS and NRAS with RRAS1, RRAS2, RRAS3. Strikingly, the binding affinities between these two groups of RAS subfamilies are indeed different.

## Discussion

Since the discovery of the first RAS effector [49,50,51,52], inhibition of RAS signaling by blocking RAS-effector interactions has been an ever-evolving and challenging venture [53,54,55,56]. Biochemical and biophysical studies providing insights into the interaction of the downstream effectors with RAS proteins and their variants established the basic principles for drug design and development [31,43,53,57,58]. There is, however, a quite significant gap in our understanding of how RAS proteins specifically bind to, and activate, their diverse effectors. Rigorous understanding of this RAS-effector interplay would require an investigation of larger fragments or full-length effector proteins that was so far been accomplished in only a few studies [36,59,60]. For several reasons, isolated effector domains have been used in the vast majority of biochemical and structural studies for the investigation of their interactions with RAS proteins, predominantly with HRAS (Table 1 and S1 Table). However, interaction characteristics obtained for the same proteins differ considerably. For example, K_d_ values for the interaction of HRAS-GTP with CRAF or RALGDS vary from 5 to 330 nM and 80 nM to 39 μM, respectively (Table 1). Another major difference of more than two orders of magnitude was observed for the interaction between RRAS1 and CRAF. Such a large variation of K_d_ values (summarized in Table 1), which in addition have been determined by different groups using different methods and experimental conditions, made a comprehensive analysis of sequence-structure-function relationships practically impossible. Thus, we have quantitatively analyzed the interaction between five effector domains and five RAS proteins, covering for the first time RRAS2, under the same conditions (Table 2).

Our measurements reveal that the RAS isoforms (HRAS, KRAS and NRAS) behave similarly toward each effector but very differently as compared to RRAS isoforms (RRAS1 and RRAS2), in spite of their high sequence identity. A previous study has reported that RAS isoforms much more strongly activate the MAPK pathway *via* the RAF kinase as compared to RRAS isoforms [60]. These data are consistent with K_d_ values determined in this study for RAS (ranging 0.048 to 0.142 μM) and RRAS (2.29 to 4.09 μM) isoforms. Notably, RRAS isoforms bind, except for PLCε, similarly to all tested effector domains with an up to 4-fold difference in binding affinities compare to RAS isoforms. Interestingly, they significantly interacted with PI3Kα but not with PLCε (Table 2), which is in agreement with the cell-based data reported previously [60].

In particular, the RAS isoforms, which exhibit high selectivity for CRAF followed by RASSF5, RALGDS and PLCε, appeared not to retain affinity for PI3Kα. It could be argued that the isolated RB domain of PI3Kα (consisting of the amino acids 169–301) may lack additional binding determinants, when compared to the 50-fold higher affinity obtained with the isolated RB domain of PI3Kγ (amino acids 144–1102) (Tables 1 and 2) [36]. A recent cell-based study has shown that RB domain of PI3Kα (aa 127–314) is sufficient to bind to ERAS, a newly discovered member of the RAS family, but obviously not to HRAS [5,61]. However, the immunoprecipitation studies have revealed the endogenous PI3K isoforms α and γ interact with almost same affinity with both ERAS and HRAS [5]. These data suggest that RB domain of PI3K is sufficient for a tight interaction with ERAS but clearly requires additional capacity to properly associate with HRAS. Sequence deviations in effector binding regions may be critical for determining the minimal binding regions of RAS/effectors. It is, therefore, hypothesized that ERAS and RRAS isoforms but not RAS isoforms efficiently interact with RB domain of PI3Ks and that RAS isoforms need a second binding region or alternatively a scaffold protein.

Considering the affinities of RAS isoforms compared to RRAS isoforms, these are very similar for both groups regardless of the effector protein. Correspondingly, the RAS isoforms have identical effector binding regions and RRAS isoforms, also including RRAS3, revealed a very high sequence identity in these regions (S3 Fig). Considering differences in affinities between them, residues outside the interacting interface may play a role in the association *via* indirect long-range interactions, electrostatic steering or allosteric modulation. However, direct interacting residues that differ between these two classes of proteins are most likely to be responsible for observed differences. Noteworthy, there are only two such amino acids in the region R3 with significant occurrence in the interaction at position 41 (Arg/Thr in RAS isoforms compared to RRAS isoforms) and 64 (Tyr/Phe). R41 in RAS isoforms interacts favorably with asparagine and aspartic acid in CRAF respectively RASSF5, most likely stabilizing the high affinity interactions with the effector proteins. These interactions appear to be much weaker if Arg-41 is replaced by a threonine in RRAS isoforms. This explains, thus, huge differences in K_d_ between the RAS isoforms and the RRAS isoforms. The same arginine does not make such favorable contact with RALGDS or PLCε, contributing to lower affinities. Its interaction with counter residues in PI3K is loose in all analyzed complexes corresponding to higher K_d_ values for this effector. Interaction at this spot may determine effector selectivity between these isoforms, as confirmed for ERAS that has a tryptophan (Trp-79) at the corresponding position of Arg-41 in HRAS and has exhibited a higher selectivity for PI3K than CRAF [61]. Another crucial hotspot at position 64 of the RAS proteins very likely also plays an important role in the interaction with effectors. In accordance with the interaction matrix, it is in the vicinity of residues at effector positions 57 and 71, respectively. The mode of interaction between these residues, however, is not pronounced as in the case of Arg-41. Substitution of Tyr-64 for Phenylalanine may have very diverse impacts on the binding affinity.

The RB and RA domains share higher sequence homologies if they are aligned individually. However, there is no common consensus sequence for RAS binding if they are aligned together, particularly in the RAS binding regions R1 to R5 (S2 Fig; see arrowheads). Previous studies dealing with the interaction of small GTPases with their regulators have shown that there are patches of identical or highly homologous hotspots on both sides of protein surfaces that interact with each other [62,63,64]. Such interaction is evolutionary conserved and responsible for the recognition of counter proteins. Our finding that there is no patch of identical amino acids in RAS effector proteins (Fig 4 and S2 Fig) seemed to break this rule. However, intermolecular β-sheet interactions between RAS proteins and their effectors are conserved and seem to supply the role of such critical patch (or in this special case, a stretch) of homologous amino acid residues. The analysis of complex structures showed that these interactions, covered by the recognition region R1 in the interaction matrix, are prevalent and occur in almost all structures. A β-sheet homodimer interface has been recently reported for the structures of KRAS-GTP that overlaps the binding site of the effectors within R1 [65]. Therefore, we have analyzed the proximity of effector binding residues in different RAS isoforms in the same way as of residues involved in β-sheet interactions and summarized the results as matrices (Fig 4A and S5 Fig). Introduction of four different interaction types in the matrix with high scores that separated main-chain and side-chain RAS-effector interactions allowed a detailed inspection of the central R1 region. Strikingly, there are three hotspots, which largely undergo main-chain/main-chain interactions (Glu-37 of RAS proteins with effector residues at position 68 and 69, respectively Asp-38 with residues at position 67; S5 Fig). These observations confirm the central role of R1 in the association of RAS proteins with their effectors and strongly suggest that the main-chain/main-chain interactions within this region are crucial for the recognition of these classes of proteins. Finally, we note that interactions in R1 also dependent, to a certain extent, on side chains of accompanying amino acids. They indirectly support the formation of β-sheet on both sides of complexes. However, they also utilize their side chains in another intramolecular interactions significantly contributing to the complex formation. In this way, Asp-38 interacts *via* its side chain exclusively with the effector residues at positions 68 and 69 within R1. Side chains of Glu-37 and Ile-37 undergo contacts with residues at positions 57 and 59 outside of the effector β-strand within the region R3. On the effector side of complexes, there are only two positions that contain identical or highly homologous amino acids, namely the position 59 and 84 (Fig 4A). In both cases they are populated by positively charged residues, with exception of PLCε that has a Gln at position 59. These residues interact with negatively charged residues on RAS proteins (Glu-37 and Asp-33) and strongly contribute to the formation of complexes. However, no unique and/or particular residue of effectors can be considered to cause the overall differences observed for their association with RAS proteins. Effector interacting residues are so variable at almost all interacting spots that only their concerted action is likely to explain the observed diversity.

Previous studies have shown that RAS variants (at residues Thr-35, Glu-37, Asp-38 and Tyr-40 and including also residues mentioned above) preferentially interact with some effectors but not others [39,40,41,42]. However, to date there is no clear explanation for the variable selections of these mutants of RAS by specific effectors. The invariant Thr-35 of RAS was not located in one of the three main regions in the matrix as it is mainly involved in RAS structure and does not directly interact with RAF1. However, Spoerner and colleagues have shown that T35S mutation drastically reduces HRAS affinity for effectors, including CRAF-RB (60-fold) and RALGDS-RA (>100-fold) [66]. They suggest that minor changes, such as truncating Thr-35 by a methyl group, strongly affect dynamic behavior of the switch 1 region and, in turn, its interaction with effectors. However, an early cell-based study has shown that HRAS T35S mutant interacts only with CRAF but not PI3K, BYR2, RALGDS or RASSF5, and activates the MAPK pathway [39]. One explanation may be that Gal1 scaffolds the HRAS^T35S^-CRAF [67]. On the other hand, the E37G mutation results in loss of PI3K and CRAF binding, but is able to interact with RA domain-containing effectors, such as RALGDS, RASSF5 and BYR2 [39]. Our interaction matrix shows contacts between E37G of HRAS and positively charged residues 61 and 69, and main-chain interactions with residue 69, and 70 of effectors. D38A mutation has been shown to retain CRAF binding but to lose interaction with PI3K, RALGDS and RASSF5 [42,68]. Among different effector binding mutants, Y40C selectively activates PI3K but is unable to activate other effectors, such as RAF1, RALGDS, RASSF5 and BYR2 [69]. HRAS^G12V/Y40C^ and HRAS^G12V/E37G^ have been reported to cooperatively induce cell transformation *via* PI3K and RALGDS, respectively, but not *via* CRAF [40]. Vandal and colleagues have observed that KRAS^G12V/Y40C^-PI3K has the largest impact on an increase in tumor size whereas KRAS^G12V/E38G^-CRAF resulted in a decrease in tumor size but an increase of the number of tumors when combined with BRAF^V600E^ [70]. Being central elements of R1, R3 and R4, our analysis not only confirms a prominent role of Glu-37, Asp-38 and Tyr-40 in effector binding but also gives hints for the mode of their interaction, which relies on the main-chain-main-chain interaction. As this interaction is largely independent of associated side chains, it can be considered as conserved in effectors. Consequently, it supplies the role of homologous residues found to be essential for the recognition of regulator proteins by Rho GTPases. Hence, we state that these RAS residues are responsible with their main-chain atoms for the recognition of effectors. On the other hand, side chains of these residues are still influential on the binding with effectors, either indirectly by affecting the structure of RAS switch I or directly by interacting with effector residues within the regions R3 and R4 of our interaction matrix.

In conclusion, our data collectively support previous observations that the specificity in the signaling properties and biological functions of the various RAS proteins arises from the specific combination of effector pathways they regulate in each cell type. Considering the identity of interacting residues of different types of isoforms, a uniform association of RAS isoforms or rather RRAS isoforms can be expected with a particular effector. This raises the questions of how does the cell selects between respective RAS proteins and maintains respective effector activation. There are several review articles illustrating the current state of the art regarding the activation mechanism of various effectors [9,11,12,13,21,71,72,73]. HRAS, KRAS and NRAS exhibit remarkable differences beyond their common interaction interfaces for regulators and effectors [74,75,76], especially at their C-terminal hypervariable region (S3 Fig), which has different features, including protein-protein interaction [77,78]. An interesting issue, which is increasingly appreciated, is a RAS-membrane interaction that appears to generate RAS isoform specificity with respect to effector interactions [79,80,81]. This is likely achieved by RAS-specific scaffold proteins, including CaM, GAL1, GAL3, IQGAPs, NPM1, NCL, SHOC2/SUR8 [78,82], which may modulate isoform specificity at specific site of the cell. Hence, elucidation of the RAS signal transduction requires not only RAS-effector interactions but also additional structures and interplay of multiprotein complexes [25]. Another critical aspect is sorting/trafficking of the isoforms [83,84] that has recently been shown to be highly specific for the respective RAS proteins and dependents on specific posttranslational modifications, including prenylation and acylation [85,86], phosphorylation [87,88], ubiquitination [89,90,91,92] and acetylation [93,94,95]. Similar characteristics have been reported for the RRAS isoforms, including protein-protein interaction required for subcellular localization, e.g., at focal adhesion or recycling endosomes,[96,97], and posttranslational modifications [98,99,100]. In addition, they contain extended N-termini (S3 Fig) that have been shown to be critical for RRAS1 in cell migration [101]. The N-terminus of ERAS, which undergoes multiple interaction with other proteins (Nakhaeizadeh *et al*., unpublished), contains (like RRAS1) putative SH3-binding motifs. These motifs may provide additional mechanisms for sorting and trafficking to specific subcellular sites.

An issue, that remained to be elucidated in more detail, is the mechanism of effector activation. Notably, identification of additional components of the RAS signal transduction is a critical step towards understanding the relationship between the RAS proteins and the selective activation of respective effectors. Functional reconstitution of RAS interaction networks by using appropriate liposomes and full-length effector proteins may eventually provide fundamental insights into the functional characterization of multiprotein complexes of RAS and the complete identification of regulatory mechanisms.

## Supporting Information

S1 Table Published structures of the RAS and Effector protein complexes. (DOCX)

S1 Fig Equilibrium dissociation constants for RAS-effector interaction. Fluorescence polarization experiments were conducted to determine the dissociation constants (K_d_) by titrating mGppNHp-bound, active forms of RAS proteins (1 μM, respectively) with increasing concentrations of the respective effector domains, as indicated. The y-axis represents fluorescence polarization and the x-axis the concentration of the effector domain as MBP fusion proteins in μM. Evaluated equilibrium K_d_ values are illustrated as bar charts in Fig 2 and summarized in Table 2.
(DOCX)

S2 Fig Sequence Alignment of the RAS effector domains. The overall amino acid alignment of RB and RA domains (A) was adjusted with structure alignment to increase the identity score. The latter was clearly increased when we separated RB domains of RAF isoforms (B) and the catalytic subunits of PI3K isoforms (C) from the RA domains (D). The five regions, described in Fig 3, are highlighted as arrowheads: R1 in red, R2 in green, R3 in blue, R4 in orange and R5 in purple. The secondary structure elements, the α helices and β sheets, from the RA domains were deduced from the crystal structures of HRAS complexes with RALGDS (PDB code: 1LFD) [37], RASSF5 (PDB code: 3DDC) [117], PLCε (PDB code: 2C5L) [34], and GRB14 (PDB code: 4K81) [118], respectively.
(DOCX)

S3 Fig Overall sequence comparison of human RAS proteins. Multiple amino acid sequence alignment of RAS proteins with high similarities has been determined by ClustalW. Interaction regions, R1 to R5, at interface with the RB and RA effector domains are illustrated by arrowhead (color-coding is the same as in Fig 4: R1 in red; R2 in green; R3 in blue; R4 in purple; R4 in orange). The secondary structure elements, the α helices and β sheets, of the G domain were deduced from the HRAS crystal structure (PDB code: 5P21) [119]. G1 to G5 boxes indicate the presence of five essential GDP/GTP binding (G) motifs. The three amino acid deviations between RAS and RRAS isoforms that are critical selectivity-determining residues for effector binding are highlighted in red.
(DOCX)

S4 Fig Known structures of the RAS-effector complexes. Nine structures of RAS-effector domain complexes were found in a PDB search, including HRAS-CRAF-RB (PDB code: 4g0n, 4G3X, 3kud), HRAS-BYR2-RB (PDB code: 1k8r), RAP1A-CRAF-RB (PDB code: 1GUA), KRAS-ARAF-RB (PDB code: 2mse), HRAS-RALGDS (PDB code: 1lfd), HRAS-PI3Kγ (PDB code: 1he8), HRAS-PLCε (PDB code: 2c5l), HRAS-RASSF (PDB code: 3ddc), HRAS-GRAB14 (PDB code: 4k81). An overlaid structure in ribbon presentation (central panel) illustrates the overall contacts of these structures (see also Fig 3). The contact sites (with distances of 4 Å or less) were calculated by Pymol and colored in white. RAS proteins are shown in orchid and the effector domains in olive as indicated.
(DOCX)

S5 Fig Intermolecular β sheet-β sheet interactions covered by the recognition region R1. Intermolecular β sheet interactions between RAS proteins and their effectors is covered by the recognition region R1 in the interaction matrix, which is launched to demonstrate interaction residues in all available structures. Left and upper panels comprises the amino acid sequence alignment of RAS and effector proteins, respectively. Each element corresponds a possible interaction of RAS (row) and effectors (column) residues. Besides, each element involves four sub-elements, which show a combination of main-chain and side-chain interactions, as indicated. Main-chain–main-chain contacts are shown in red.
(DOCX)

S1 File Supporting References. (DOCX)

## References

[1] Wittinghofer A, Herrmann C (1995) Ras-effector interactions, the problem of specificity. FEBS Lett 369: 52–56. 7641884 10.1016/0014-5793(95)00667-x

[2] Gutierrez-Erlandsson S, Herrero-Vidal P, Fernandez-Alfara M, Hernandez-Garcia S, Gonzalo-Flores S, Mudarra-Rubio A, et al (2013) R-RAS2 overexpression in tumors of the human central nervous system. Mol Cancer 12: 127 10.1186/1476-4598-12-127 24148564 PMC3900289

[3] Karnoub AE, Weinberg RA (2008) Ras oncogenes: split personalities. Nature reviews Molecular cell biology 9: 517–531. 10.1038/nrm2438 18568040 PMC3915522

[4] Herrmann C (2003) Ras-effector interactions: after one decade. Curr Opin Struct Biol 13: 122–129. 12581669 10.1016/s0959-440x(02)00007-6

[5] Nakhaei-Rad S, Nakhaeizadeh H, Gotze S, Kordes C, Sawitza I, Hoffmann MJ, et al (2016) The Role of Embryonic Stem Cell-expressed RAS (ERAS) in the Maintenance of Quiescent Hepatic Stellate Cells. The Journal of biological chemistry 291: 8399–8413. 10.1074/jbc.M115.700088 26884329 PMC4861415

[6] Castellano E, Downward J (2010) Role of RAS in the regulation of PI 3-kinase. Curr Top Microbiol Immunol 346: 143–169. 10.1007/82_2010_56 20563706

[7] Chan JJ, Katan M (2013) PLCvarepsilon and the RASSF family in tumour suppression and other functions. Advances in biological regulation 53: 258–279. 10.1016/j.jbior.2013.07.008 23958207

[8] Bunney TD, Katan M (2011) PLC regulation: emerging pictures for molecular mechanisms. Trends Biochem Sci 36: 88–96. 10.1016/j.tibs.2010.08.003 20870410

[9] Ferro E, Trabalzini L (2010) RalGDS family members couple Ras to Ral signalling and that's not all. Cellular signalling 22: 1804–1810. 10.1016/j.cellsig.2010.05.010 20478380

[10] Rajalingam K, Schreck R, Rapp UR, Albert S (2007) Ras oncogenes and their downstream targets. Biochimica et biophysica acta 1773: 1177–1195. 10.1016/j.bbamcr.2007.01.012 17428555

[11] Desideri E, Cavallo AL, Baccarini M (2015) Alike but Different: RAF Paralogs and Their Signaling Outputs. Cell 161: 967–970. 10.1016/j.cell.2015.04.045 26000477

[12] Castellano E, Downward J (2011) RAS Interaction with PI3K: More Than Just Another Effector Pathway. Genes & cancer 2: 261–274. 21779497 10.1177/1947601911408079 PMC3128635

[13] Gentry LR, Martin TD, Reiner DJ, Der CJ (2014) Ral small GTPase signaling and oncogenesis: More than just 15minutes of fame. Biochimica et biophysica acta 1843: 2976–2988. 10.1016/j.bbamcr.2014.09.004 25219551 PMC4201770

[14] Bunney TD, Katan M (2006) Phospholipase C epsilon: linking second messengers and small GTPases. Trends Cell Biol 16: 640–648. 10.1016/j.tcb.2006.10.007 17085049

[15] Feig LA, Buchsbaum RJ (2002) Cell signaling: life or death decisions of ras proteins. Curr Biol 12: R259–261. 11937045 10.1016/s0960-9822(02)00787-x

[16] Bryant KL, Mancias JD, Kimmelman AC, Der CJ (2014) KRAS: feeding pancreatic cancer proliferation. Trends Biochem Sci 39: 91–100. 10.1016/j.tibs.2013.12.004 24388967 PMC3955735

[17] Cirstea IC, Gremer L, Dvorsky R, Zhang SC, Piekorz RP, Zenker M, et al (2013) Diverging gain-of-function mechanisms of two novel KRAS mutations associated with Noonan and cardio-facio-cutaneous syndromes. Human molecular genetics 22: 262–270. 10.1093/hmg/dds426 23059812

[18] Gremer L, Merbitz-Zahradnik T, Dvorsky R, Cirstea IC, Kratz CP, Zenker M, et al (2011) Germline KRAS mutations cause aberrant biochemical and physical properties leading to developmental disorders. Human mutation 32: 33–43. 10.1002/humu.21377 20949621 PMC3117284

[19] Lissewski C, Kant SG, Stark Z, Schanze I, Zenker M (2015) Copy number variants including RAS pathway genes-How much RASopathy is in the phenotype? Am J Med Genet A 167A: 2685–2690. 10.1002/ajmg.a.37155 25974318

[20] McCormick F (2015) KRAS as a Therapeutic Target. Clin Cancer Res 21: 1797–1801. 10.1158/1078-0432.CCR-14-2662 25878360 PMC4407814

[21] Donninger H, Schmidt ML, Mezzanotte J, Barnoud T, Clark GJ (2016) Ras signaling through RASSF proteins. Seminars in cell & developmental biology. 10.1016/j.semcdb.2016.06.007 PMC5034565 27288568

[22] Dhillon AS, Hagan S, Rath O, Kolch W (2007) MAP kinase signalling pathways in cancer. Oncogene 26: 3279–3290. 10.1038/sj.onc.1210421 17496922

[23] Repasky GA, Chenette EJ, Der CJ (2004) Renewing the conspiracy theory debate: does Raf function alone to mediate Ras oncogenesis? Trends Cell Biol 14: 639–647. 10.1016/j.tcb.2004.09.014 15519853

[24] Wohlgemuth S, Kiel C, Kramer A, Serrano L, Wittinghofer F, Herrmann C (2005) Recognizing and defining true Ras binding domains I: biochemical analysis. Journal of molecular biology 348: 741–758. 10.1016/j.jmb.2005.02.048 15826668

[25] Nussinov R, Tsai CJ, Muratcioglu S, Jang H, Gursoy A, Keskin O (2015) Principles of K-Ras effector organization and the role of oncogenic K-Ras in cancer initiation through G1 cell cycle deregulation. Expert Rev Proteomics 12: 669–682. 10.1586/14789450.2015.1100079 26496174

[26] Ahearn IM, Haigis K, Bar-Sagi D, Philips MR (2012) Regulating the regulator: post-translational modification of RAS. Nature reviews Molecular cell biology 13: 39–51. 10.1038/nrm3255 PMC3879958 22189424

[27] Hennig A, Markwart R, Esparza-Franco MA, Ladds G, Rubio I (2015) Ras activation revisited: role of GEF and GAP systems. Biol Chem 396: 831–848. 10.1515/hsz-2014-0257 25781681

[28] Fischer A, Hekman M, Kuhlmann J, Rubio I, Wiese S, Rapp UR (2007) B- and C-RAF display essential differences in their binding to Ras: the isotype-specific N terminus of B-RAF facilitates Ras binding. The Journal of biological chemistry 282: 26503–26516. 10.1074/jbc.M607458200 17635919

[29] Mott HR, Owen D (2015) Structures of Ras superfamily effector complexes: What have we learnt in two decades? Critical Reviews in Biochemistry and Molecular Biology 50: 85–133. 10.3109/10409238.2014.999191 25830673

[30] Vetter IR, Wittinghofer A (2001) The guanine nucleotide-binding switch in three dimensions. Science 294: 1299–1304. 10.1126/science.1062023 11701921

[31] Athuluri-Divakar SK, Vasquez-Del Carpio R, Dutta K, Baker SJ, Cosenza SC, Basu I, et al (2016) A Small Molecule RAS-Mimetic Disrupts RAS Association with Effector Proteins to Block Signaling. Cell 165: 643–655. 10.1016/j.cell.2016.03.045 27104980 PMC5006944

[32] Thapar R, Williams JG, Campbell SL (2004) NMR characterization of full-length farnesylated and non-farnesylated H-Ras and its implications for Raf activation. Journal of molecular biology 343: 1391–1408. 10.1016/j.jmb.2004.08.106 15491620

[33] Drugan JK, Khosravi-Far R, White MA, Der CJ, Sung YJ, Hwang YW, et al (1996) Ras interaction with two distinct binding domains in Raf-1 may be required for Ras transformation. The Journal of biological chemistry 271: 233–237. 8550565 10.1074/jbc.271.1.233

[34] Bunney TD, Harris R, Gandarillas NL, Josephs MB, Roe SM, Sorli SC, et al (2006) Structural and Mechanistic Insights into Ras Association Domains of Phospholipase C Epsilon. Molecular Cell 21: 495–507. 10.1016/j.molcel.2006.01.008 16483931

[35] Stieglitz B, Bee C, Schwarz D, Yildiz O, Moshnikova A, Khokhlatchev A, et al (2008) Novel type of Ras effector interaction established between tumour suppressor NORE1A and Ras switch II. EMBO J 27: 1995–2005. 10.1038/emboj.2008.125 18596699 PMC2486280

[36] Pacold ME, Suire S, Perisic O, Lara-Gonzalez S, Davis CT, Walker EH, et al (2000) Crystal structure and functional analysis of Ras binding to its effector phosphoinositide 3-kinase gamma. Cell 103: 931–943. 11136978 10.1016/s0092-8674(00)00196-3

[37] Huang L, Hofer F, Martin GS, Kim SH (1998) Structural basis for the interaction of Ras with RalGDS. Nat Struct Biol 5: 422–426. 9628477 10.1038/nsb0698-422

[38] Nassar N, Horn G, Herrmann C, Scherer A, McCormick F, Wittinghofer A (1995) The 2.2 A crystal structure of the Ras-binding domain of the serine/threonine kinase c-Raf1 in complex with Rap1A and a GTP analogue. Nature 375: 554–560. 10.1038/375554a0 7791872

[39] White MA, Nicolette C, Minden A, Polverino A, Van Aelst L, Karin M, et al (1995) Multiple Ras functions can contribute to mammalian cell transformation. Cell 80: 533–541. 7867061 10.1016/0092-8674(95)90507-3

[40] Khosravi-Far R, White MA, Westwick JK, Solski PA, Chrzanowska-Wodnicka M, Van Aelst L, et al (1996) Oncogenic Ras activation of Raf/mitogen-activated protein kinase-independent pathways is sufficient to cause tumorigenic transformation. Mol Cell Biol 16: 3923–3933. 8668210 10.1128/mcb.16.7.3923 PMC231389

[41] Khwaja A, Rodriguez-Viciana P, Wennstrom S, Warne PH, Downward J (1997) Matrix adhesion and Ras transformation both activate a phosphoinositide 3-OH kinase and protein kinase B/Akt cellular survival pathway. EMBO J 16: 2783–2793. 10.1093/emboj/16.10.2783 9184223 PMC1169887

[42] Vavvas D, Li X, Avruch J, Zhang XF (1998) Identification of Nore1 as a potential Ras effector. J Biol Chem 273: 5439–5442. 9488663 10.1074/jbc.273.10.5439

[43] Erijman A, Shifman JM (2016) RAS/Effector Interactions from Structural and Biophysical Perspective. Mini Rev Med Chem 16: 370–375. 26423700 10.2174/1389557515666151001141838

[44] Hall TA (1999) BioEdit: a user-friendly biological sequence alignment editor and analysis program for Windows 95/98/NT. Nucleic Acids Symposium Series 41: 95–98.

[45] Pettersen EF, Goddard TD, Huang CC, Couch GS, Greenblatt DM, Meng EC, et al (2004) UCSF Chimera—A visualization system for exploratory research and analysis. Journal of Computational Chemistry 25: 1605–1612. 10.1002/jcc.20084 15264254

[46] Cock PJ, Antao T, Chang JT, Chapman BA, Cox CJ, Dalke A, et al (2009) Biopython: freely available Python tools for computational molecular biology and bioinformatics. Bioinformatics 25: 1422–1423. 10.1093/bioinformatics/btp163 19304878 PMC2682512

[47] DeLano WL (2002) The PyMOL Molecular Graphics System.

[48] Raines RT (2015) Fluorescence polarization assay to quantify protein-protein interactions: an update. Methods Mol Biol 1278: 323–327. 10.1007/978-1-4939-2425-7_19 25859958

[49] Kolch W, Heidecker G, Lloyd P, Rapp UR (1991) Raf-1 protein kinase is required for growth of induced NIH/3T3 cells. Nature 349: 426–428. 10.1038/349426a0 1992343

[50] Warne PH, Viciana PR, Downward J (1993) Direct interaction of Ras and the amino-terminal region of Raf-1 in vitro. Nature 364: 352–355. 10.1038/364352a0 8332195

[51] Zhang XF, Settleman J, Kyriakis JM, Takeuchi-Suzuki E, Elledge SJ, Marshall MS, et al (1993) Normal and oncogenic p21ras proteins bind to the amino-terminal regulatory domain of c-Raf-1. Nature 364: 308–313. 10.1038/364308a0 8332187

[52] Moodie SA, Willumsen BM, Weber MJ, Wolfman A (1993) Complexes of Ras.GTP with Raf-1 and mitogen-activated protein kinase kinase. Science 260: 1658–1661. 8503013 10.1126/science.8503013

[53] Lu S, Jang H, Gu S, Zhang J, Nussinov R (2016) Drugging Ras GTPase: a comprehensive mechanistic and signaling structural view. Chem Soc Rev. 10.1039/c5cs00911a PMC5021603 27396271

[54] Shima F, Yoshikawa Y, Matsumoto S, Kataoka T (2013) Discovery of small-molecule Ras inhibitors that display antitumor activity by interfering with Ras.GTP-effector interaction. Enzymes 34 Pt. B: 1–23. 25034098 10.1016/B978-0-12-420146-0.00001-9

[55] Beeram M, Patnaik A, Rowinsky EK (2003) Regulation of c-Raf-1: therapeutic implications. Clin Adv Hematol Oncol 1: 476–481. 16258435

[56] Beeram M, Patnaik A, Rowinsky EK (2005) Raf: a strategic target for therapeutic development against cancer. J Clin Oncol 23: 6771–6790. 10.1200/JCO.2005.08.036 16170185

[57] Ostrem JM, Shokat KM (2016) Direct small-molecule inhibitors of KRAS: from structural insights to mechanism-based design. Nat Rev Drug Discov. 10.1038/nrd.2016.139 27469033

[58] Cromm PM, Spiegel J, Grossmann TN, Waldmann H (2015) Direct Modulation of Small GTPase Activity and Function. Angew Chem Int Ed Engl 54: 13516–13537. 10.1002/anie.201504357 26470842

[59] Kurig B, Shymanets A, Bohnacker T, Prajwal, Brock C, Ahmadian MR, et al (2009) Ras is an indispensable coregulator of the class IB phosphoinositide 3-kinase p87/p110gamma. Proc Natl Acad Sci U S A 106: 20312–20317. 10.1073/pnas.0905506106 19906996 PMC2787109

[60] Rodriguez-Viciana P, Sabatier C, McCormick F (2004) Signaling specificity by Ras family GTPases is determined by the full spectrum of effectors they regulate. Mol Cell Biol 24: 4943–4954. 10.1128/MCB.24.11.4943-4954.2004 15143186 PMC416418

[61] Nakhaei-Rad S, Nakhaeizadeh H, Kordes C, Cirstea IC, Schmick M, Dvorsky R, et al (2015) The Function of Embryonic Stem Cell-expressed RAS (E-RAS), a Unique RAS Family Member, Correlates with Its Additional Motifs and Its Structural Properties. The Journal of biological chemistry 290: 15892–15903. 10.1074/jbc.M115.640607 25940089 PMC4505495

[62] Dvorsky R, Ahmadian MR (2004) Always look on the bright site of Rho: structural implications for a conserved intermolecular interface. EMBO Rep 5: 1130–1136. 10.1038/sj.embor.7400293 15577926 PMC1299188

[63] Jaiswal M, Dvorsky R, Ahmadian MR (2013) Deciphering the molecular and functional basis of Dbl family proteins: a novel systematic approach toward classification of selective activation of the Rho family proteins. The Journal of biological chemistry 288: 4486–4500. 10.1074/jbc.M112.429746 23255595 PMC3567697

[64] Amin E, Jaiswal M, Derewenda U, Reis K, Nouri K, Koessmeier KT, et al (2016) Deciphering the molecular and functional basis of RhoGAP family proteins: A systematic approach towards selective inactivation of Rho family proteins. The Journal of biological chemistry. 10.1074/jbc.M116.736967 PMC5034035 27481945

[65] Muratcioglu S, Chavan TS, Freed BC, Jang H, Khavrutskii L, Freed RN, et al (2015) GTP-Dependent K-Ras Dimerization. Structure 23: 1325–1335. 10.1016/j.str.2015.04.019 26051715 PMC4497850

[66] Spoerner M, Herrmann C, Vetter IR, Kalbitzer HR, Wittinghofer A (2001) Dynamic properties of the Ras switch I region and its importance for binding to effectors. Proc Natl Acad Sci U S A 98: 4944–4949. 10.1073/pnas.081441398 11320243 PMC33143

[67] Elad-Sfadia G, Haklai R, Ballan E, Gabius HJ, Kloog Y (2002) Galectin-1 augments Ras activation and diverts Ras signals to Raf-1 at the expense of phosphoinositide 3-kinase. J Biol Chem 277: 37169–37175. 10.1074/jbc.M205698200 12149263

[68] Katz ME, McCormick F (1997) Signal transduction from multiple Ras effectors. Curr Opin Genet Dev 7: 75–79. 9024640 10.1016/s0959-437x(97)80112-8

[69] Rodriguez-Viciana P, Warne PH, Khwaja A, Marte BM, Pappin D, Das P, et al (1997) Role of phosphoinositide 3-OH kinase in cell transformation and control of the actin cytoskeleton by Ras. Cell 89: 457–467. 9150145 10.1016/s0092-8674(00)80226-3

[70] Vandal G, Geiling B, Dankort D (2014) Ras effector mutant expression suggest a negative regulator inhibits lung tumor formation. PloS one 9: e84745 10.1371/journal.pone.0084745 24489653 PMC3904846

[71] Wing MR, Bourdon DM, Harden TK (2003) PLC-epsilon: a shared effector protein in Ras-, Rho-, and G alpha beta gamma-mediated signaling. Mol Interv 3: 273–280. 10.1124/mi.3.5.273 14993441

[72] Wellbrock C, Karasarides M, Marais R (2004) The RAF proteins take centre stage. Nature reviews Molecular cell biology 5: 875–885. 10.1038/nrm1498 15520807

[73] Baljuls A, Kholodenko BN, Kolch W (2013) It takes two to tango—signalling by dimeric Raf kinases. Mol Biosyst 9: 551–558. 10.1039/c2mb25393c 23212737

[74] Ahmadian MR, Hoffmann U, Goody RS, Wittinghofer A (1997) Individual rate constants for the interaction of Ras proteins with GTPase-activating proteins determined by fluorescence spectroscopy. Biochemistry 36: 4535–4541. 10.1021/bi962556y 9109662

[75] Scheffzek K, Ahmadian MR, Wittinghofer A (1998) GTPase-activating proteins: helping hands to complement an active site. Trends Biochem Sci 23: 257–262. 9697416 10.1016/s0968-0004(98)01224-9

[76] Castellano E, Santos E (2011) Functional specificity of ras isoforms: so similar but so different. Genes & cancer 2: 216–231. 21779495 10.1177/1947601911408081 PMC3128637

[77] Chavan TS, Jang H, Khavrutskii L, Abraham SJ, Banerjee A, Freed BC, et al (2015) High-Affinity Interaction of the K-Ras4B Hypervariable Region with the Ras Active Site. Biophys J 109: 2602–2613. 10.1016/j.bpj.2015.09.034 26682817 PMC4699860

[78] Abraham SJ, Nolet RP, Calvert RJ, Anderson LM, Gaponenko V (2009) The hypervariable region of K-Ras4B is responsible for its specific interactions with calmodulin. Biochemistry 48: 7575–7583. 10.1021/bi900769j 19583261 PMC2729490

[79] Parker JA, Mattos C (2015) The Ras-Membrane Interface: Isoform-specific Differences in The Catalytic Domain. Molecular cancer research: MCR 13: 595–603. 10.1158/1541-7786.MCR-14-0535 25566993

[80] Abankwa D, Hanzal-Bayer M, Ariotti N, Plowman SJ, Gorfe AA, Parton RG, et al (2008) A novel switch region regulates H-ras membrane orientation and signal output. EMBO J 27: 727–735. 10.1038/emboj.2008.10 18273062 PMC2265749

[81] Mazhab-Jafari MT, Marshall CB, Smith MJ, Gasmi-Seabrook GM, Stathopulos PB, Inagaki F, et al (2015) Oncogenic and RASopathy-associated K-RAS mutations relieve membrane-dependent occlusion of the effector-binding site. Proc Natl Acad Sci U S A 112: 6625–6630. 10.1073/pnas.1419895112 25941399 PMC4450377

[82] Rodriguez-Viciana P, Oses-Prieto J, Burlingame A, Fried M, McCormick F (2006) A phosphatase holoenzyme comprised of Shoc2/Sur8 and the catalytic subunit of PP1 functions as an M-Ras effector to modulate Raf activity. Molecular cell 22: 217–230. 10.1016/j.molcel.2006.03.027 16630891

[83] Zhou M, Wiener H, Su W, Zhou Y, Liot C, Ahearn I, et al (2016) VPS35 binds farnesylated N-Ras in the cytosol to regulate N-Ras trafficking. The Journal of cell biology 214: 445–458. 10.1083/jcb.201604061 27502489 PMC4987297

[84] Zheng ZY, Cheng CM, Fu XR, Chen LY, Xu L, Terrillon S, et al (2012) CHMP6 and VPS4A mediate the recycling of Ras to the plasma membrane to promote growth factor signaling. Oncogene 31: 4630–4638. 10.1038/onc.2011.607 22231449 PMC3326214

[85] Jang H, Abraham SJ, Chavan TS, Hitchinson B, Khavrutskii L, Tarasova NI, et al (2015) Mechanisms of membrane binding of small GTPase K-Ras4B farnesylated hypervariable region. The Journal of biological chemistry 290: 9465–9477. 10.1074/jbc.M114.620724 25713064 PMC4392252

[86] Lynch SJ, Snitkin H, Gumper I, Philips MR, Sabatini D, Pellicer A (2015) The differential palmitoylation states of N-Ras and H-Ras determine their distinct Golgi subcompartment localizations. J Cell Physiol 230: 610–619. 10.1002/jcp.24779 25158650 PMC4269384

[87] Bivona TG, Quatela SE, Bodemann BO, Ahearn IM, Soskis MJ, Mor A, et al (2006) PKC regulates a farnesyl-electrostatic switch on K-Ras that promotes its association with Bcl-XL on mitochondria and induces apoptosis. Molecular cell 21: 481–493. 10.1016/j.molcel.2006.01.012 16483930

[88] Sung PJ, Tsai FD, Vais H, Court H, Yang J, Fehrenbacher N, et al (2013) Phosphorylated K-Ras limits cell survival by blocking Bcl-xL sensitization of inositol trisphosphate receptors. Proc Natl Acad Sci U S A 110: 20593–20598. 10.1073/pnas.1306431110 24297914 PMC3870738

[89] Wang MT, Holderfield M, Galeas J, Delrosario R, To MD, Balmain A, et al (2015) K-Ras Promotes Tumorigenicity through Suppression of Non-canonical Wnt Signaling. Cell 163: 1237–1251. 10.1016/j.cell.2015.10.041 26590425

[90] Rodriguez-Viciana P, McCormick F (2006) Ras ubiquitination: coupling spatial sorting and signal transmission. Cancer Cell 9: 243–244. 10.1016/j.ccr.2006.03.025 16616329

[91] Jura N, Scotto-Lavino E, Sobczyk A, Bar-Sagi D (2006) Differential modification of Ras proteins by ubiquitination. Molecular cell 21: 679–687. 10.1016/j.molcel.2006.02.011 16507365

[92] de la Vega M, Burrows JF, McFarlane C, Govender U, Scott CJ, Johnston JA (2010) The deubiquitinating enzyme USP17 blocks N-Ras membrane trafficking and activation but leaves K-Ras unaffected. The Journal of biological chemistry 285: 12028–12036. 10.1074/jbc.M109.081448 20147298 PMC2852940

[93] Yang MH, Laurent G, Bause AS, Spang R, German N, Haigis MC, et al (2013) HDAC6 and SIRT2 regulate the acetylation state and oncogenic activity of mutant K-RAS. Molecular cancer research: MCR 11: 1072–1077. 10.1158/1541-7786.MCR-13-0040-T 23723075 PMC3778089

[94] Knyphausen P, Lang F, Baldus L, Extra A, Lammers M (2016) Insights into K-Ras 4B regulation by post-translational lysine acetylation. Biol Chem. 10.1515/hsz-2016-0118 27176741

[95] Yang MH, Nickerson S, Kim ET, Liot C, Laurent G, Spang R, et al (2012) Regulation of RAS oncogenicity by acetylation. Proc Natl Acad Sci U S A 109: 10843–10848. 10.1073/pnas.1201487109 22711838 PMC3390846

[96] Wurtzel JG, Lee S, Singhal SS, Awasthi S, Ginsberg MH, Goldfinger LE (2015) RLIP76 regulates Arf6-dependent cell spreading and migration by linking ARNO with activated R-Ras at recycling endosomes. Biochemical and biophysical research communications 467: 785–791. 10.1016/j.bbrc.2015.10.064 26498519 PMC4644438

[97] Furuhjelm J, Peranen J (2003) The C-terminal end of R-Ras contains a focal adhesion targeting signal. J Cell Sci 116: 3729–3738. 10.1242/jcs.00689 12890755

[98] Berzat AC, Brady DC, Fiordalisi JJ, Cox AD (2006) Using inhibitors of prenylation to block localization and transforming activity. Methods Enzymol 407: 575–597. 10.1016/S0076-6879(05)07046-1 16757354

[99] Oertli B, Han J, Marte BM, Sethi T, Downward J, Ginsberg M, et al (2000) The effector loop and prenylation site of R-Ras are involved in the regulation of integrin function. Oncogene 19: 4961–4969. 10.1038/sj.onc.1203876 11042683

[100] Calvo F, Crespo P (2009) Structural and spatial determinants regulating TC21 activation by RasGRF family nucleotide exchange factors. Molecular biology of the cell 20: 4289–4302. 10.1091/mbc.E09-03-0212 19692568 PMC2762140

[101] Holly SP, Larson MK, Parise LV (2005) The unique N-terminus of R-ras is required for Rac activation and precise regulation of cell migration. Molecular biology of the cell 16: 2458–2469. 10.1091/mbc.E03-12-0917 15772154 PMC1087249

[102] Herrmann C, Martin GA, Wittinghofer A (1995) Quantitative analysis of the complex between p21ras and the Ras-binding domain of the human Raf-1 protein kinase. The Journal of biological chemistry 270: 2901–2905. 7852367 10.1074/jbc.270.7.2901

[103] Gorman C, Skinner RH, Skelly JV, Neidle S, Lowe PN (1996) Equilibrium and kinetic measurements reveal rapidly reversible binding of Ras to Raf. The Journal of biological chemistry 271: 6713–6719. 8636091 10.1074/jbc.271.12.6713

[104] Hwang MC, Sung YJ, Hwang YW (1996) The differential effects of the Gly-60 to Ala mutation on the interaction of H-Ras p21 with different downstream targets. The Journal of biological chemistry 271: 8196–8202. 8626511 10.1074/jbc.271.14.8196

[105] Linnemann T, Geyer M, Jaitner BK, Block C, Kalbitzer HR, Wittinghofer A, et al (1999) Thermodynamic and kinetic characterization of the interaction between the Ras binding domain of AF6 and members of the Ras subfamily. The Journal of biological chemistry 274: 13556–13562. 10224125 10.1074/jbc.274.19.13556

[106] Boettner B, Herrmann C, Van Aelst L (2001) Ras and Rap1 interaction with AF-6 effector target. Methods Enzymol 332: 151–168. 11305093 10.1016/s0076-6879(01)32199-7

[107] Linnemann T, Zheng YH, Mandic R, Peterlin BM (2002) Interaction between Nef and phosphatidylinositol-3-kinase leads to activation of p21-activated kinase and increased production of HIV. Virology 294: 246–255. 10.1006/viro.2002.1365 12009866

[108] Gremer L, De Luca A, Merbitz-Zahradnik T, Dallapiccola B, Morlot S, Tartaglia M, et al (2010) Duplication of Glu37 in the switch I region of HRAS impairs effector/GAP binding and underlies Costello syndrome by promoting enhanced growth factor-dependent MAPK and AKT activation. Human molecular genetics 19: 790–802. 10.1093/hmg/ddp548 19995790

[109] Sydor JR, Engelhard M, Wittinghofer A, Goody RS, Herrmann C (1998) Transient kinetic studies on the interaction of Ras and the Ras-binding domain of c-Raf-1 reveal rapid equilibration of the complex. Biochemistry 37: 14292–14299. 10.1021/bi980764f 9760267

[110] Sydor JR, Seidel RP, Goody RS, Engelhard M (1999) Cell-free synthesis of the Ras-binding domain of c-Raf-1: binding studies to fluorescently labelled H-ras. FEBS Lett 452: 375–378. 10386625 10.1016/s0014-5793(99)00633-x

[111] Vetter IR, Linnemann T, Wohlgemuth S, Geyer M, Kalbitzer HR, Herrmann C, et al (1999) Structural and biochemical analysis of Ras-effector signaling via RalGDS. FEBS Lett 451: 175–180. 10371160 10.1016/s0014-5793(99)00555-4

[112] Rudolph MG, Linnemann T, Grunewald P, Wittinghofer A, Vetter IR, Herrmann C (2001) Thermodynamics of Ras/effector and Cdc42/effector interactions probed by isothermal titration calorimetry. The Journal of biological chemistry 276: 23914–23921. 10.1074/jbc.M011600200 11292826

[113] Harjes E, Harjes S, Wohlgemuth S, Muller KH, Krieger E, Herrmann C, et al (2006) GTP-Ras disrupts the intramolecular complex of C1 and RA domains of Nore1. Structure 14: 881–888. 10.1016/j.str.2006.03.008 16698549

[114] Hunter JC, Manandhar A, Carrasco MA, Gurbani D, Gondi S, Westover KD (2015) Biochemical and Structural Analysis of Common Cancer-Associated KRAS Mutations. Molecular cancer research: MCR 13: 1325–1335. 10.1158/1541-7786.MCR-15-0203 26037647

[115] Flex E, Jaiswal M, Pantaleoni F, Martinelli S, Strullu M, Fansa EK, et al (2014) Activating mutations in RRAS underlie a phenotype within the RASopathy spectrum and contribute to leukaemogenesis. Human molecular genetics 23: 4315–4327. 10.1093/hmg/ddu148 24705357 PMC4103678

[116] Herrmann C, Horn G, Spaargaren M, Wittinghofer A (1996) Differential interaction of the ras family GTP-binding proteins H-Ras, Rap1A, and R-Ras with the putative effector molecules Raf kinase and Ral-guanine nucleotide exchange factor. The Journal of biological chemistry 271: 6794–6800. 8636102 10.1074/jbc.271.12.6794

[117] Stieglitz B, Bee C, Schwarz D, Yildiz Ö, Moshnikova A, Khokhlatchev A, et al (2008) Novel type of Ras effector interaction established between tumour suppressor NORE1A and Ras switch II. The EMBO journal 27: 1995–2005. 10.1038/emboj.2008.125 18596699 PMC2486280

[118] Qamra R, Hubbard SR (2013) Structural Basis for the Interaction of the Adaptor Protein Grb14 with Activated Ras. PLoS One 8: e72473 10.1371/journal.pone.0072473 23967305 PMC3742580

[119] Pai EF, Kabsch W, Krengel U, Holmes KC, John J, Wittinghofer A (1989) Structure of the guanine-nucleotide-binding domain of the Ha-ras oncogene product p21 in the triphosphate conformation. Nature 341: 209–214. 10.1038/341209a0 2476675

