# Supplementary material for: The RAS-Effector Interface: Isoform-Specific Differences in the Effector Binding Regions
Source: PLoS One. 2016 Dec 9;11(12):e0167145. doi: 10.1371/journal.pone.0167145 (PMC5147862; doi:10.1371/journal.pone.0167145)
Supplement: S1 Table — (DOCX) [file pone.0167145.s001.docx]

**Supporting information**

**The RAS-effector interface: Isoform-specific differences in the effector binding regions**

H. Nakhaeizadeh, E. Amin, S. Nakhaei-Rad, R. Dvorsky, M. R. Ahmadian

Institute of Biochemistry and Molecular Biology II,

Medical Faculty of the Heinrich-Heine University, Düsseldorf, Germany

**S1 Table. . Published structures of the RAS and Effector protein complexes.**

| Proteins | PDB code | Resolution (Å) | Reference |
| --- | --- | --- | --- |
| RAP1A(E30D/K31E)-GppNHp-CRAF-RB | 1GUA | 2.0 | [[1](#_ENREF_1)] |
| HRAS-GppNHp-RALGDS | 1LFD | 2.1 | [[2](#_ENREF_2)] |
| HRAS(G12V)-GppNHp-PI3Kγ-RB(V223K/V326A) | 1HE8 | 3.0 | [[3](#_ENREF_3)] |
| HRAS-GDP-CRAF-RB(A85K) | 3KUD | 2.15 | [[4](#_ENREF_4)] |
| HRAS**-**GppNHp**-**Byr2-RB | 1K8R | 3.0 | [[5](#_ENREF_5)] |
| HRAS(G12V)-GTP-PLCε(Y2176L) | 2C5L | 1.9 | [[6](#_ENREF_6)] |
| HRAS(D30E/E31K)-GppNHp-RASSF5-RA (L285M/K302D) | 3DDC | 1.8 | [[7](#_ENREF_7)] |
| HRAS(G12V)-GTP**·**GRAB14-RA/PH (K272A/E273A) | 4K81 | 2.4 | [[8](#_ENREF_8)] |
| HRAS-GppNHp-CRAF-RB | 4G0N | 2.45 | [[9](#_ENREF_9)] |
| HRAS(Q61L)-GppNHp-CRAF-RB | 4G3X | 3.25 | [[9](#_ENREF_9)] |
| KRAS-GppNHp-ARAF-RB | 2MSE | NMR | [[10](#_ENREF_10)] |
