# Supplementary material for: The RAS-Effector Interface: Isoform-Specific Differences in the Effector Binding Regions
Source: PLoS One. 2016 Dec 9;11(12):e0167145. doi: 10.1371/journal.pone.0167145 (PMC5147862; doi:10.1371/journal.pone.0167145)
Supplement: S1 Fig — Fluorescence polarization experiments were conducted to determine the dissociation constants (Kd) by titrating mGppNHp-bound, active forms of RAS proteins (1 μM, respectively) with increasing concentrations of the respective effector domains, as indicated. The y-axis represents fluorescence polarization and the x-axis the concentration of the effector domain as MBP fusion proteins in μM. Evaluated equilibrium Kd values are illustrated as bar charts in Fig 2 and summarized in Table 2. (DOCX) [file pone.0167145.s002.docx]

**Supporting information**

**The RAS-effector interface: Isoform-specific differences in the effector binding regions**

H. Nakhaeizadeh, E. Amin, S. Nakhaei-Rad, R. Dvorsky, M. R. Ahmadian

Institute of Biochemistry and Molecular Biology II,

Medical Faculty of the Heinrich-Heine University, Düsseldorf, Germany


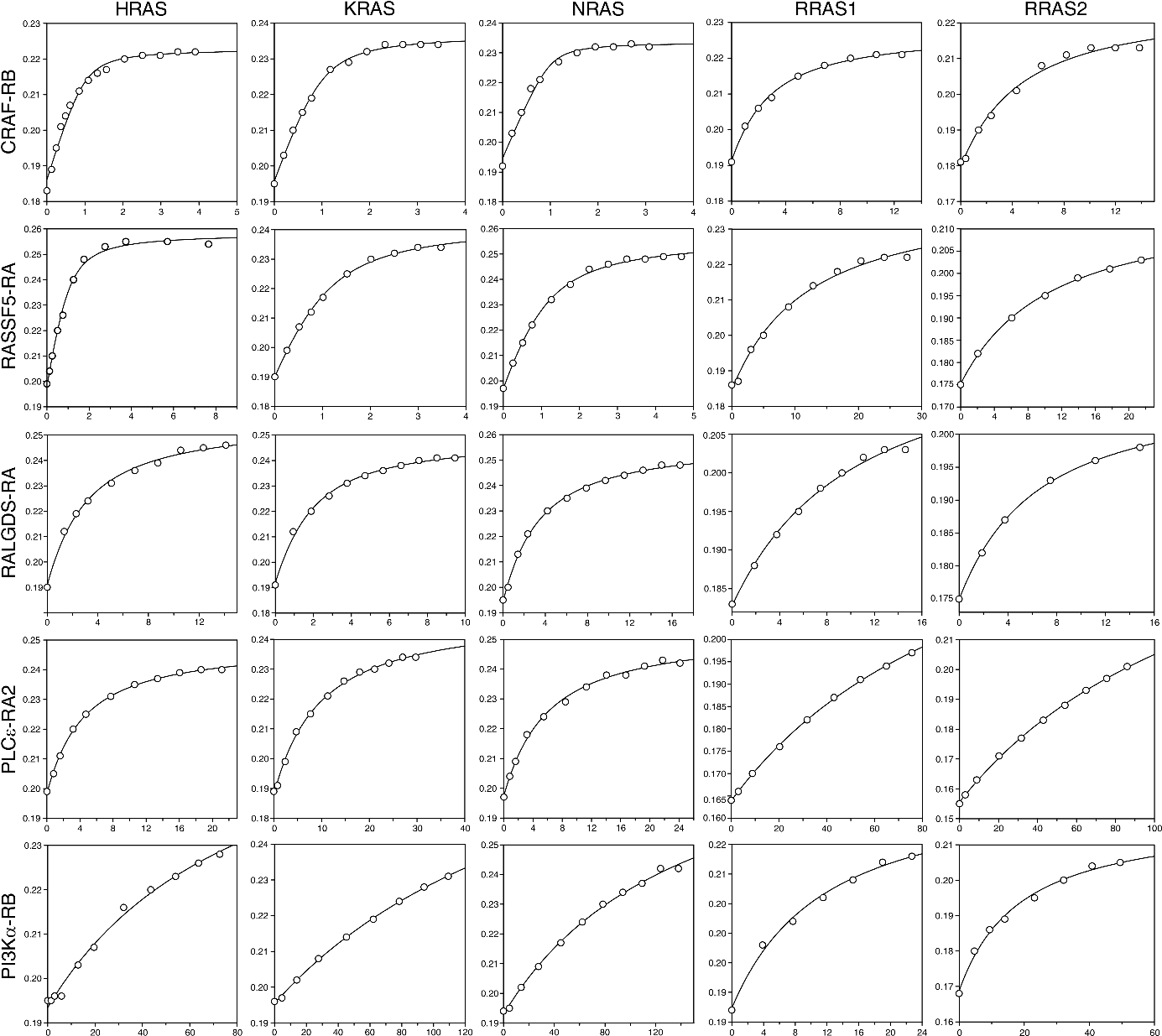


**S1 Fig. Equilibrium dissociation constants for RAS-effector interaction**. Fluorescence polarization experiments were conducted to determine the dissociation constants (K_d_) by titrating mGppNHp-bound, active forms of RAS proteins (1 µM, respectively) with increasing concentrations of the respective effector domains, as indicated. The y-axis represents fluorescence polarization and the x-axis the concentration of the effector domain as MBP fusion proteins in µM. Evaluated equilibrium K_d_ values are illustrated as bar charts in Figure 2 and summarized in Table 2.
