# Supplementary material for: The RAS-Effector Interface: Isoform-Specific Differences in the Effector Binding Regions
Source: PLoS One. 2016 Dec 9;11(12):e0167145. doi: 10.1371/journal.pone.0167145 (PMC5147862; doi:10.1371/journal.pone.0167145)
Supplement: S2 Fig — The overall amino acid alignment of RB and RA domains (A) was adjusted with structure alignment to increase the identity score. The latter was clearly increased when we separated RB domains of RAF isoforms (B) and the catalytic subunits of PI3K isoforms (C) from the RA domains (D). The five regions, described in Fig 3, are highlighted as arrowheads: R1 in red, R2 in green, R3 in blue, R4 in orange and R5 in purple. The secondary structure elements, the α helices and β sheets, from the RA domains were deduced from the crystal structures of HRAS complexes with RALGDS (PDB code: 1LFD) [37], RASSF5 (PDB code: 3DDC) [117], PLCε (PDB code: 2C5L) [34], and GRB14 (PDB code: 4K81) [118], respectively. (DOCX) [file pone.0167145.s003.docx]

**Supporting information**

**The RAS-effector interface: Isoform-specific differences in the effector binding regions**

H. Nakhaeizadeh, E. Amin, S. Nakhaei-Rad, R. Dvorsky, M. R. Ahmadian

Institute of Biochemistry and Molecular Biology II,

Medical Faculty of the Heinrich-Heine University, Düsseldorf, Germany


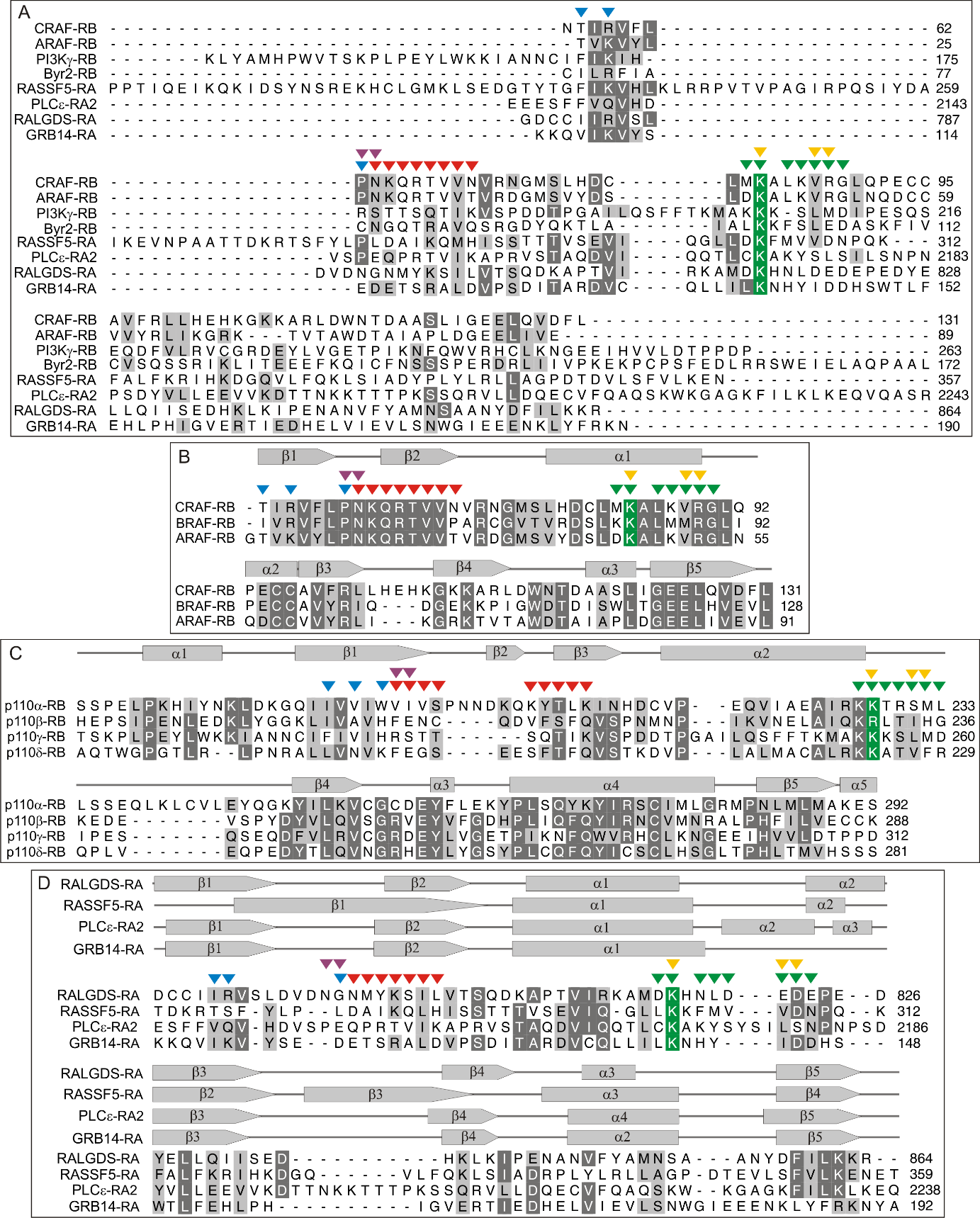


**S2 Fig. Sequence Alignment of the RAS effector domains.** The overall amino acid alignment of RB and RA domains (A) was adjusted with structure alignment to increase the identity score. The latter was clearly increased when we separated RB domains of RAF isoforms (B) and the catalytic subunits of PI3K isoforms (C) from the RA domains (D). The five regions, described in Figure 3, are highlighted as arrowheads: R1 in red, R2 in green, R3 in blue, R4 in orange and R5 in purple. The secondary structure elements, the α helices and β sheets, from the RA domains were deduced from the crystal structures of HRAS complexes with RALGDS (PDB code: 1LFD) [[2](#_ENREF_2)], RASSF5 (PDB code: 3DDC ) [[7](#_ENREF_7)], PLCε (PDB code: 2C5L) [[6](#_ENREF_6)], and GRB14 (PDB code: 4K81) [[8](#_ENREF_8)], respectively.
