# Supplementary material for: The RAS-Effector Interface: Isoform-Specific Differences in the Effector Binding Regions
Source: PLoS One. 2016 Dec 9;11(12):e0167145. doi: 10.1371/journal.pone.0167145 (PMC5147862; doi:10.1371/journal.pone.0167145)
Supplement: S3 Fig — Multiple amino acid sequence alignment of RAS proteins with high similarities has been determined by ClustalW. Interaction regions, R1 to R5, at interface with the RB and RA effector domains are illustrated by arrowhead (color-coding is the same as in Fig 4: R1 in red; R2 in green; R3 in blue; R4 in purple; R4 in orange). The secondary structure elements, the α helices and β sheets, of the G domain were deduced from the HRAS crystal structure (PDB code: 5P21) [119]. G1 to G5 boxes indicate the presence of five essential GDP/GTP binding (G) motifs. The three amino acid deviations between RAS and RRAS isoforms that are critical selectivity-determining residues for effector binding are highlighted in red. (DOCX) [file pone.0167145.s004.docx]

**Supporting information**

**The RAS-effector interface: Isoform-specific differences in the effector binding regions**

H. Nakhaeizadeh, E. Amin, S. Nakhaei-Rad, R. Dvorsky, M. R. Ahmadian

Institute of Biochemistry and Molecular Biology II,

Medical Faculty of the Heinrich-Heine University, Düsseldorf, Germany


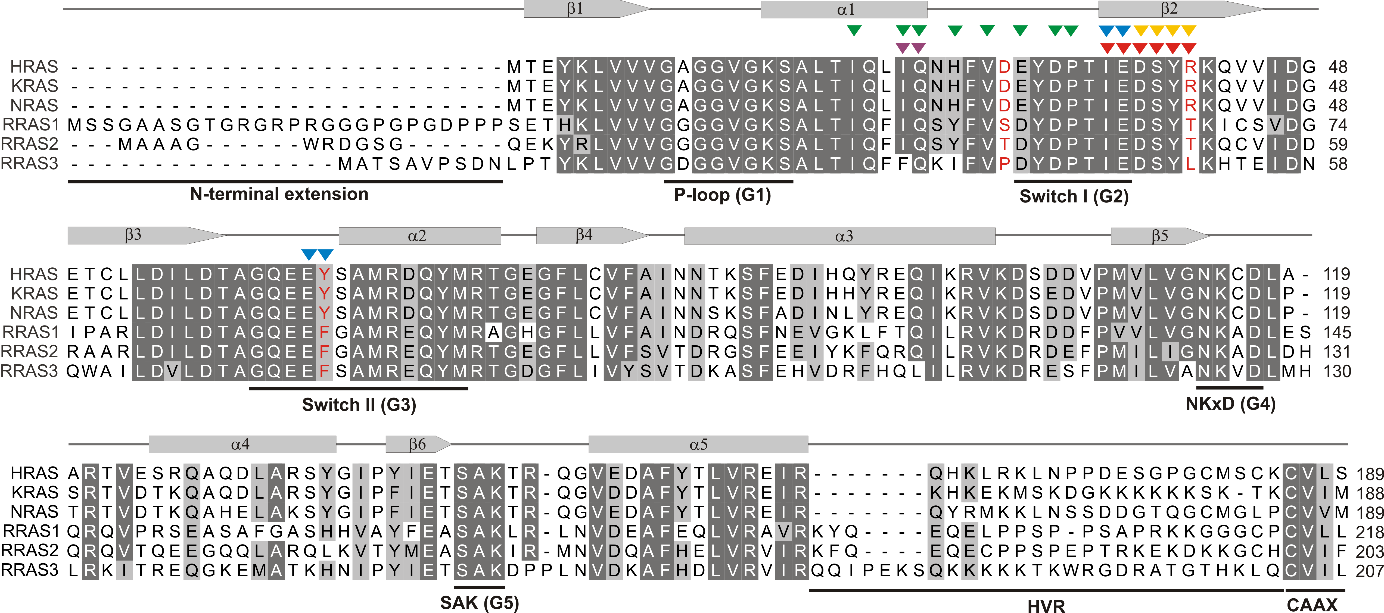


**S3 Fig. Overall sequence comparison of human RAS proteins.** Multiple amino acid sequence alignment of RAS proteins with high similarities has been determined by ClustalW. Interaction regions, R1 to R5, at interface with the RB and RA effector domains are illustrated by arrowhead (color-coding is the same as in Fig. 4: R1 in red; R2 in green; R3 in blue; R4 in purple; R4 in orange). The secondary structure elements, the α helices and β sheets, of the G domain were deduced from the HRAS crystal structure (PDB code: 5P21) [[11](#_ENREF_11)]. G1 to G5 boxes indicate the presence of five essential GDP/GTP binding (G) motifs. The three amino acid deviations between RAS and RRAS isoforms that are critical selectivity-determining residues for effector binding are highlighted in red.
