# Supplementary material for: The RAS-Effector Interface: Isoform-Specific Differences in the Effector Binding Regions
Source: PLoS One. 2016 Dec 9;11(12):e0167145. doi: 10.1371/journal.pone.0167145 (PMC5147862; doi:10.1371/journal.pone.0167145)
Supplement: S4 Fig — Nine structures of RAS-effector domain complexes were found in a PDB search, including HRAS-CRAF-RB (PDB code: 4g0n, 4G3X, 3kud), HRAS-BYR2-RB (PDB code: 1k8r), RAP1A-CRAF-RB (PDB code: 1GUA), KRAS-ARAF-RB (PDB code: 2mse), HRAS-RALGDS (PDB code: 1lfd), HRAS-PI3Kγ (PDB code: 1he8), HRAS-PLCε (PDB code: 2c5l), HRAS-RASSF (PDB code: 3ddc), HRAS-GRAB14 (PDB code: 4k81). An overlaid structure in ribbon presentation (central panel) illustrates the overall contacts of these structures (see also Fig 3). The contact sites (with distances of 4 Å or less) were calculated by Pymol and colored in white. RAS proteins are shown in orchid and the effector domains in olive as indicated. (DOCX) [file pone.0167145.s005.docx]

**Supporting information**

**The RAS-effector interface: Isoform-specific differences in the effector binding regions**

H. Nakhaeizadeh, E. Amin, S. Nakhaei-Rad, R. Dvorsky, M. R. Ahmadian

Institute of Biochemistry and Molecular Biology II,

Medical Faculty of the Heinrich-Heine University, Düsseldorf, Germany

**
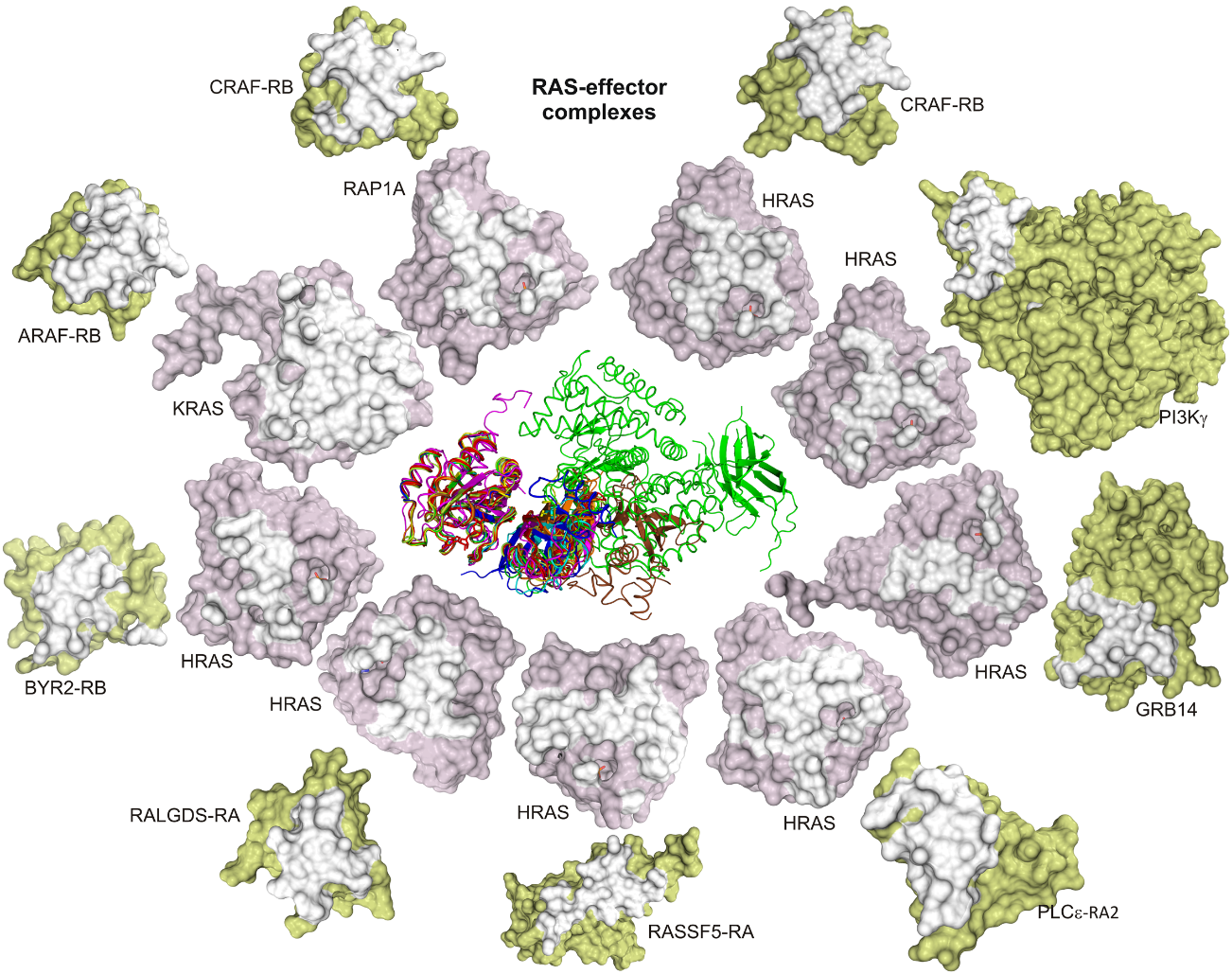
**

**S4 Fig. Known structures of the RAS-effector complexes.** Nine structures of RAS-effector domain complexes were found in a PDB search, including HRAS-CRAF-RB (PDB code: 4g0n, 4G3X, 3kud), HRAS-BYR2-RB (PDB code: 1k8r), RAP1A-CRAF-RB (PDB code: 1GUA), KRAS-ARAF-RB (PDB code: 2mse), HRAS-RALGDS (PDB code: 1lfd), HRAS-PI3Kγ (PDB code: 1he8), HRAS-PLCε (PDB code: 2c5l), HRAS-RASSF (PDB code: 3ddc), HRAS-GRAB14 (PDB code: 4k81). An overlaid structure in ribbon presentation (central panel) illustrates the overall contacts of these structures (see also Figure 3). The contact sites (with distances of 4 Å or less) were calculated by Pymol and colored in white. RAS proteins are shown in orchid and the effector domains in olive as indicated.
