# Supplementary material for: The RAS-Effector Interface: Isoform-Specific Differences in the Effector Binding Regions
Source: PLoS One. 2016 Dec 9;11(12):e0167145. doi: 10.1371/journal.pone.0167145 (PMC5147862; doi:10.1371/journal.pone.0167145)
Supplement: S5 Fig — Intermolecular β sheet interactions between RAS proteins and their effectors is covered by the recognition region R1 in the interaction matrix, which is launched to demonstrate interaction residues in all available structures. Left and upper panels comprises the amino acid sequence alignment of RAS and effector proteins, respectively. Each element corresponds a possible interaction of RAS (row) and effectors (column) residues. Besides, each element involves four sub-elements, which show a combination of main-chain and side-chain interactions, as indicated. Main-chain–main-chain contacts are shown in red. (DOCX) [file pone.0167145.s006.docx]

**Supporting information**

**The RAS-effector interface: Isoform-specific differences in the effector binding regions**

H. Nakhaeizadeh, E. Amin, S. Nakhaei-Rad, R. Dvorsky, M. R. Ahmadian

Institute of Biochemistry and Molecular Biology II,

Medical Faculty of the Heinrich-Heine University, Düsseldorf, Germany


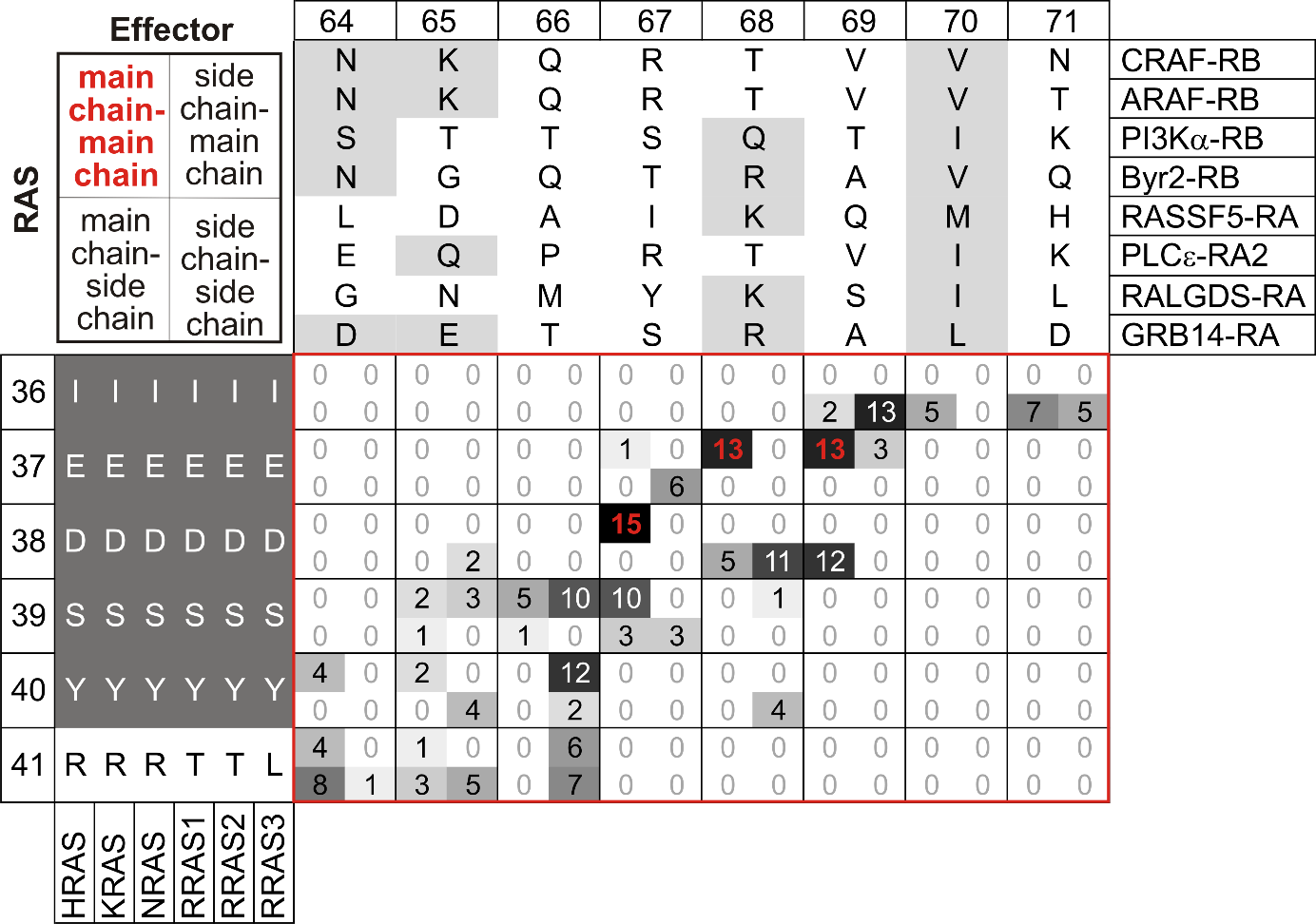


**S5 Fig. Intermolecular β sheet‑β sheet interactions covered by the recognition region R1.** Intermolecular β sheet interactions between RAS proteins and their effectors is covered by the recognition region R1 in the interaction matrix, which is launched to demonstrate interaction residues in all available structures. Left and upper panels comprises the amino acid sequence alignment of RAS and effector proteins, respectively. Each element corresponds a possible interaction of RAS (row) and effectors (column) residues. Besides, each element involves four sub-elements, which show a combination of main-chain and side-chain interactions, as indicated. Main-chain–main-chain contacts are shown in red.
