# Supplementary material for: The RAS-Effector Interface: Isoform-Specific Differences in the Effector Binding Regions
Source: PLoS One. 2016 Dec 9;11(12):e0167145. doi: 10.1371/journal.pone.0167145 (PMC5147862; doi:10.1371/journal.pone.0167145)
Supplement: S1 File — (DOCX) [file pone.0167145.s007.docx]

**Supporting information**

**The RAS-effector interface: Isoform-specific differences in the effector binding regions**

H. Nakhaeizadeh, E. Amin, S. Nakhaei-Rad, R. Dvorsky, M. R. Ahmadian

Institute of Biochemistry and Molecular Biology II,

Medical Faculty of the Heinrich-Heine University, Düsseldorf, Germany

**S1 file. Supporting References**

1. Nassar N, Horn G, Herrmann C, Block C, Janknecht R, et al. (1996) Ras/Rap effector specificity determined by charge reversal. Nat Struct Biol 3: 723-729.

2. Huang L, Hofer F, Martin GS, Kim SH (1998) Structural basis for the interaction of Ras with RalGDS. Nat Struct Biol 5: 422-426.

3. Pacold ME, Suire S, Perisic O, Lara-Gonzalez S, Davis CT, et al. (2000) Crystal structure and functional analysis of Ras binding to its effector phosphoinositide 3-kinase gamma. Cell 103: 931-943.

4. Filchtinski D, Sharabi O, Rüppel A, Vetter IR, Herrmann C, et al. (2010) What Makes Ras an Efficient Molecular Switch: A Computational, Biophysical, and Structural Study of Ras-GDP Interactions with Mutants of Raf. Journal of Molecular Biology 399: 422-435.

5. Scheffzek K, Grunewald P, Wohlgemuth S, Kabsch W, Tu H, et al. (2001) The Ras-Byr2RBD complex: structural basis for Ras effector recognition in yeast. Structure 9: 1043-1050.

6. Bunney TD, Harris R, Gandarillas NL, Josephs MB, Roe SM, et al. (2006) Structural and Mechanistic Insights into Ras Association Domains of Phospholipase C Epsilon. Molecular Cell 21: 495-507.

7. Stieglitz B, Bee C, Schwarz D, Yildiz Ö, Moshnikova A, et al. (2008) Novel type of Ras effector interaction established between tumour suppressor NORE1A and Ras switch II. The EMBO journal 27: 1995-2005.

8. Qamra R, Hubbard SR (2013) Structural Basis for the Interaction of the Adaptor Protein Grb14 with Activated Ras. PLoS One 8: e72473.

9. Fetics Susan K, Guterres H, Kearney Bradley M, Buhrman G, Ma B, et al. (2015) Allosteric Effects of the Oncogenic RasQ61L Mutant on Raf-RBD. Structure 23: 505-516.

10. Mazhab-Jafari MT, Marshall CB, Smith MJ, Gasmi-Seabrook GMC, Stathopulos PB, et al. (2015) Oncogenic and RASopathy-associated K-RAS mutations relieve membrane-dependent occlusion of the effector-binding site. Proceedings of the National Academy of Sciences 112: 6625-6630.

11. Pai EF, Kabsch W, Krengel U, Holmes KC, John J, et al. (1989) Structure of the guanine-nucleotide-binding domain of the Ha-ras oncogene product p21 in the triphosphate conformation. Nature 341: 209-214.
